# The transcription factor E2A drives neural differentiation in pluripotent cells

**DOI:** 10.1242/dev.184093

**Published:** 2020-06-22

**Authors:** Chandrika Rao, Mattias Malaguti, John O. Mason, Sally Lowell

**Affiliations:** 1 MRC Centre for Regenerative Medicine, Institute for Stem Cell Research, School of Biological Sciences, University of Edinburgh, 5 Little France Drive, Edinburgh EH16 4UU, UK; 2 Centre for Discovery Brain Sciences, University of Edinburgh, 15 George Square, Edinburgh EH8 9XD, UK; 3 Simons Initiative for the Developing Brain, Hugh Robson Building, George Square, Edinburgh EH8 9XD, UK

**Keywords:** E2A, BHLH, Neural development, Pluripotent

## Abstract

The intrinsic mechanisms that link extracellular signalling to the onset of neural differentiation are not well understood. In pluripotent mouse cells, BMP blocks entry into the neural lineage via transcriptional upregulation of inhibitor of differentiation (Id) factors. We have previously identified the major binding partner of Id proteins in pluripotent cells as the basic helix-loop-helix (bHLH) transcription factor (TF) E2A. Id1 can prevent E2A from forming heterodimers with bHLH TFs or from forming homodimers. Here, we show that overexpression of a forced E2A homodimer is sufficient to drive robust neural commitment in pluripotent cells, even under non-permissive conditions. Conversely, we find that E2A null cells display a defect in their neural differentiation capacity. E2A acts as an upstream activator of neural lineage genes, including *Sox1* and *Foxd4*, and as a repressor of Nodal signalling. Our results suggest a crucial role for E2A in establishing neural lineage commitment in pluripotent cells.

## Abstract (precis)

**Summary:** E2A is usually a permissive partner for instructive transcription factors. Expression and genome editing assays revealed that E2A goes it alone as a homodimer to dominantly impose neural identity on pluripotent cells.

## INTRODUCTION

Following the establishment of the primary germ layers during gastrulation, the embryonic neural plate is specified from the anterior ectoderm at approximately embryonic day (E)7.5 in a process known as neural induction (Tam and Zhou, 1996). Pluripotent embryonic stem cells (ESCs) recapitulate central features of this process when differentiated in culture (Ying et al., 2003b), providing a useful system in which to study the mechanisms guiding these initial cell fate decisions during development. Although the key extracellular signalling pathways that inhibit neural lineage commitment have long been established, less progress has been made in identifying the downstream effectors of these pathways. In particular, inhibition of BMP signalling is crucial for the establishment of the neuroectoderm (Harland, 2000; Di-Gregorio et al., 2007). The finding that BMP signalling inhibits neural differentiation via transcriptional upregulation of Id1 (Ying et al., 2003a) provides compelling evidence that Id proteins block the activity of a factor that would otherwise trigger the onset of neural commitment.

Id proteins lack a DNA-binding domain and function primarily as dominant-negative inhibitors of basic helix-loop-helix (bHLH) transcription factors (TFs), binding to and preventing them from forming functional dimers (Norton, 2000). It has previously been reported that Id1 indirectly inhibits the activity of the bHLH TF *Tcf15.* However, *Tcf15* appears to play a role in general priming for differentiation by enabling morphological changes, and does not have a specific instructive role in neural commitment (Davies et al., 2013; Lin et al., 2019 preprint). We have also found that E-cadherin (Cdh1) acts downstream of BMP to help suppress neural commitment (Malaguti et al., 2013) but it is not known if or how Id1 is mechanistically linked with this process. Id1 is also reported to block the activity of the epigenetic regulator Zrf1, preventing derepression of neural genes in ESCs (Aloia et al., 2015). Zrf1 overexpression alone, however, is not sufficient to drive expression of these genes, suggesting a requirement for additional factors to initiate neural differentiation in ESCs.

We have previously identified the alternatively spliced E2A gene products E47 and E12 as the main binding partners of Id1 in ESCs (Davies et al., 2013). E2A (also known as Tcf3 – not to be confused with Tcf7L1, which is also commonly known as Tcf3) belongs to the E-protein family of bHLH TFs, which also includes HEB (Tcf12) and E2-2 (Tcf4). E2A is able to regulate the transcription of its target genes either by homodimerisation or by heterodimerisation with class II bHLH TFs, such as the proneural factors Ascl1 and neurogenin1/2 (Murre et al., 1989). Although E2A-bHLH heterodimers are well-established regulators of several fate determination processes, including neuronal subtype specification (Imayoshi and Kageyama, 2014), E2A homodimers have only been identified to function in the context of B-cell development (Shen and Kadesch, 1995), and it is not currently known whether this homodimer could also operate to control cell fate in other contexts.

E2A knockout mouse models have thus far failed to identify any overt gastrulation defects, with a failure of B-cell specification being the only major phenotype described to date (Bain et al., 1994; Zhuang et al., 1994). More recent analysis of these models, however, has noted that knockout mice have a significantly reduced brain size compared with their wild-type counterparts (Ravanpay and Olson, 2008), suggesting that a more in-depth investigation into the role of E2A during the earlier stages of development might be required to uncover subtle neural differentiation defects. In *Xenopus* embryos, loss of E2A has been associated with the inhibition of gastrulation (Yoon et al., 2011). Additionally, E2A and HEB have been shown to be co-factors of the Nodal signalling pathway, both in human ESCs and in *Xenopus* (Yoon et al., 2011), with E2A playing a dual role to directly repress the Nodal target gene *lefty* during mesendoderm specification in *Xenopus*, while also driving the expression of dorsal cell fate genes (Wills and Baker, 2015).

In this study, we investigate a potential role for E2A homodimers in neural fate commitment. We first set out to characterise the expression of E2A during early neural differentiation by the generation of an endogenously tagged E2A-V5 ESC line. Using a gain-of-function approach, we find that overexpression of a forced E2A homodimer, but not monomer, is sufficient to drive the neural commitment of ESCs, even in the presence of serum, providing novel mechanistic insight into the molecular events unfolding downstream of Id1 during neural commitment. CRISPR/Cas9 targeting of E2A and HEB loci to generate single and double E-protein knockout ESC lines, additionally reveals that E-protein deficiency compromises the neural differentiation ability of ESCs. RNA-sequencing (RNA-seq) analysis further confirmed that E2A is positioned upstream of the expression of several neural lineage-associated genes, including *Sox1* and *Foxd4*, and might additionally play a role in suppressing Nodal signalling during neural differentiation. We therefore propose that the E2A homodimer is a key intrinsic regulator of neural fate commitment in pluripotent ESCs.

## RESULTS

### E2A is expressed heterogeneously in pluripotent ESCs and throughout neural differentiation

**Fig. 1.**
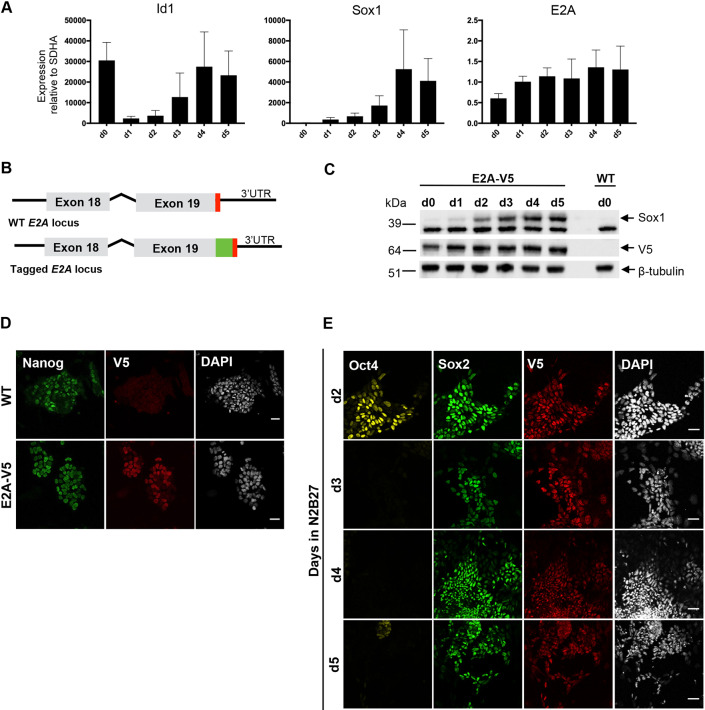
**Endogenous tagging reveals E2A is expressed heterogeneously in pluripotent cells.** (A) qRT-PCR analysis of pluripotent ESCs plated under standard neural differentiation conditions. Expression values are normalised to the housekeeping gene *Sdha*. Data are mean±s.d. *n*=3 biological replicates. (B) Schematic of wild-type (WT) and tagged *E2A* locus. The V5 epitope tag was knocked-in at the 3′ UTR of the endogenous *E2A* locus. The V5 tag is shown in green and the stop codon in red. (C) Western blot analysis of E2A-V5 cells during neural differentiation. β-Tubulin was analysed as a loading control. (D) Immunostaining of parental wild-type ESCs and epitope-tagged E2A-V5 ESCs. Cells co-stained for the V5 tag, the pluripotency marker Nanog and nuclear marker DAPI. (E) Immunostaining of E2A-V5 cells from days 2 to 5 of neural differentiation. Cells were stained for Oct4 and Sox2 to enable identification of cells committing to the neural lineage (Oct4^−^/Sox2^+^). Scale bars: 30 µm.

To characterise the expression of E2A during early neural differentiation we first examined the temporal expression of *E2A* mRNA in ESCs, plated under standard neural monolayer conditions (Ying et al., 2003b), by qRT-PCR. *Sox1* is the earliest specific marker of the neuroectoderm in mice (Pevny et al., 1998) and is therefore used to follow neural fate acquisition in ESCs. In line with previously published data (Ying et al., 2003b; Aiba et al., 2009), we observed that expression of the negative regulator of E2A, *Id1*, is rapidly downregulated at the onset of differentiation (Fig. 1A). *E2A* expression, however, remains fairly constant during this initial period. As E2A is regulated by Id1 at the protein level, rather than the transcriptional level, we generated an endogenously tagged ESC line (Fig. 1B) using CRISPR/Cas9 targeting to follow the expression of E2A protein during differentiation. Based on a strategy previously developed to tag neural stem cells with high efficiency (Dewari et al., 2018), guide RNA (sgRNA) was designed to cut proximally to the stop codon in the 3′ UTR of *E2A* (Fig. S1A), and was co-transfected into wild-type ESCs with recombinant Cas9 protein (rCas9) and a single-stranded donor DNA template (ssODN) encoding the V5 tag flanked by homology arms (Fig. S1B). Clonal lines were isolated from the bulk population and individual clones were subsequently screened by PCR genotyping (Fig. S1C), and analysed by immunostaining (Fig. 1D) for the V5 fusion protein. Sanger sequencing confirmed the error-free insertion of V5 into the C-terminus of the *E2A* locus by homology-directed repair (Fig. S1D).

We monitored the expression of the endogenously tagged E2A protein during neural differentiation using an antibody directed against the V5 tag for western blot analysis. This showed that E2A protein is expressed stably across the timecourse, with a slight increase in expression as cells exit pluripotency at day 1 (Fig. 1C), mirroring the pattern of *E2A* mRNA expression (Fig. 1A). Immunostaining of E2A-V5 cells revealed that E2A expression is heterogeneous in pluripotent ESCs on the single cell level in leukaemia inhibitory factor (LIF)/serum culture (Fig. 1D). When E2A-V5 ESCs are plated under neural differentiation conditions, E2A expression remains high and retains a heterogeneous expression pattern as cells exit the pluripotent state (Oct4^+^Sox2^−^), and progressively commit to the neural lineage (Oct4^−^Sox2^+^) (Fig. 1E).

### E2A homodimers promote neural fate commitment under self-renewal conditions

**Fig. 2.**
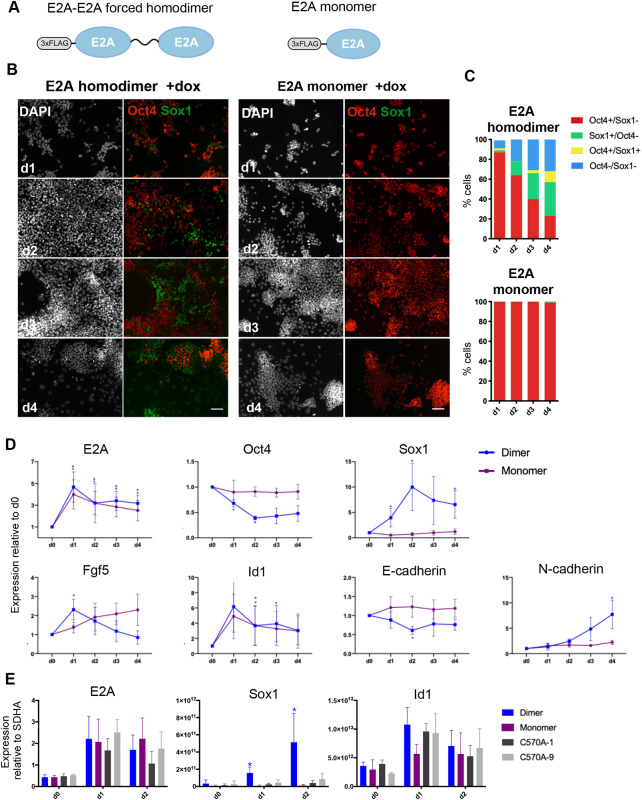
**An E2A forced homodimer drives Sox1 expression under self-renewal conditions.** (A) Schematic representation of monomeric E2A and forced E2A homodimer constructs. (B) Immunostaining of inducible E2A forced homodimers and monomers cultured in LIF/serum+dox. Scale bars: 30 µm. (C) Quantification of immunostaining for E2A monomers and homodimers cultured in LIF/serum+dox. A range of 500 to 5000 cells were manually scored for each timepoint. (D) qRT-PCR analysis of dox-inducible overexpression of E2A monomers and homodimers in LIF/serum. Expression is normalised to day 0 minus dox controls. (E) qRT-PCR analysis of two mutant forced homodimer clones (C570A-1 and C570A-9) induced in LIF/serum+dox. Expression values are normalised to the housekeeping gene *Sdha*. Data are mean±s.d., *n*=3 biological replicates. *P<0.05, two-tailed paired Student's *t*-test.

Having observed that E2A expression is dynamic during the early stages of differentiation, we next wanted to address whether E2A could play an instructive role during this cell fate transition. To determine whether E2A is sufficient to promote neural differentiation, we generated doxycycline-inducible ESC lines to overexpress either monomeric E2A or a forced E2A homodimer in which two E2A sequences are tethered by a flexible amino acid linker (Fig. 2A). This forced dimer strategy not only renders E2A more resistant to inhibition by Id but also favours the formation of E2A homodimers due to the physical proximity of the two molecules (Neuhold and Wold, 1993). FLAG-tagged E47 monomer or forced homodimer constructs were placed under the control of a tetracycline-response element and introduced into ESCs containing an inducible cassette exchange locus upstream of the HPRT gene (Iacovino et al., 2013). When expression of the two E2A constructs was induced by doxycycline (dox) in LIF/serum culture, conditions that are inhibitory for neural differentiation, we found that overexpression of the forced E2A homodimer, but not the monomer, elicited a robust upregulation of Sox1 (Fig. 2B,D; Fig. S2). Coincident with the peak of *Sox1* expression on day 2, we also observed that cells overexpressing the homodimer lost the domed colony morphology typical of ESCs, and instead spread out to cover the dish. We evaluated that 34% of cells were Sox1^+^Oct4^−^ after 4 days of culture (Fig. 2C), which was indicative of neural commitment. qRT-PCR analysis revealed that forced expression of E2A homodimers also enhanced the expression of the neural marker N-cadherin (*Cdh2*), downregulated *Cdh1* expression, and caused a transient upregulation of the epiblast marker *Fgf5* (Fig. 2D). Overexpression of both the monomeric and forced homodimer forms of E2A also appeared to stimulate a feedback response, causing upregulation of the negative regulator of E2A, *Id1*, as has been observed previously (Bhattacharya and Baker, 2011).

It has been reported that E2A homodimers are stabilised by an intermolecular disulphide bond formed between cysteine residues located in helix one of the bHLH domain (Benezra, 1994) (Fig. S3A). To confirm that it was specifically E2A homodimers, and not E2A-bHLH heterodimers, that are driving neural commitment in ESCs, we generated E2A forced dimer cell lines in which cysteine-570 is mutated to alanine in both E2A sequences (Fig. S3B) in an attempt to disrupt the formation of this covalent bond and therefore destabilise homodimer formation. These mutants are reported to retain heterodimerisation ability (Benezra, 1994). Two such mutant lines, C570A-1 and C570A-9, were stimulated with dox in LIF/serum for 2 days. qRT-PCR analysis of both mutant and control inducible lines showed that although C570A mutants were still able to upregulate *Id1*, they were no longer able to upregulate expression of *Sox1* (Fig. 2E), suggesting that this single amino acid substitution did, indeed, ablate the ability of E2A to drive differentiation. Western blot analysis of monomeric and forced E2A homodimer constructs demonstrated that expression of the forced homodimer was considerably lower than that of the monomer (Fig. S3C), ruling out the possibility that enhanced activity of the forced homodimer is explained simply by increased protein stability or the accumulation of E2A. C570A mutant lines appeared to express E2A at slightly lower levels than their wild-type homodimer counterparts. Taken together, these findings demonstrate that overexpression of E2A homodimers is sufficient to override the potent inhibitory effect of serum in non-permissive culture conditions, consistent with a role for E2A homodimers in initiating neural fate commitment.

### *E2A*^−/−^ and *E2A*^−/−^*HEB*^−/−^ knockout ESCs display compromised neural lineage commitment ability

We next set out to evaluate whether E2A is necessary for neural differentiation. To generate E2A knockout ESCs, we used CRISPR/Cas9 to target exon 3 of the *E2A* gene locus, disrupting both *E12* and *E47* transcripts. Owing to the high degree of functional compensation observed between E-protein family members (Zhuang et al., 1998), which could mask potential differentiation phenotypes, we also generated *E2A*^−/−^*HEB*^−/−^ double E-protein knockout ESC lines by using a similar strategy to target exon 9 of *HEB* in the E2A^−/−^ cell line (Fig. S4A). Targeted lines were validated by western blot analysis and sequencing of the targeted loci (Fig. S4B-F).

**Fig. 3.**
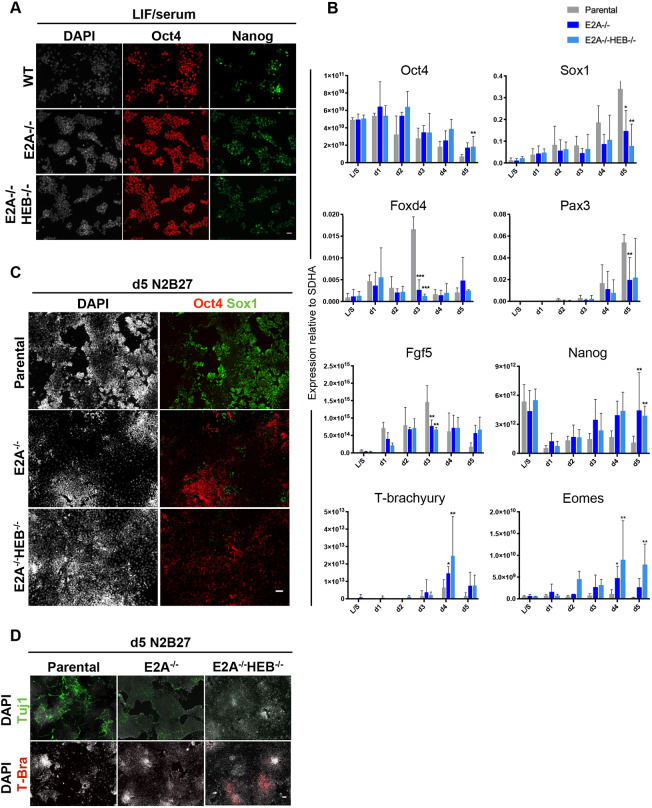
***E2A*^−/−^ and *E2A*^−/−^*HEB*^−/−^** **ESCs** **self-renew normally but display defects in early neural commitment.** (A) Immunostaining of WT, *E2A*^−/−^ and *E2A*^−/−^*HEB*^−/−^ knockout ESCs stained for pluripotency markers in LIF/serum (L/S). (B) qRT-PCR analysis of parental and knockout cell lines in L/S and differentiated in N2B27 over 5 days. Expression values are normalised to the housekeeping gene *Sdha*. Data are mean±s.d., *n*=3 biological replicates. *P<0.05, **P<0.01 and ***P<0.001, two-tailed paired Student's *t*-test. (C,D) Cells differentiated in N2B27 for 5 days stained for Oct4 and Sox1 (C), or the neuronal marker Tuj1 and the mesendodermal marker T-brachyury (T-bra). Scale bars: 30 µm.

To first determine whether E-protein knockout cells retain characteristic features of pluripotent stem cells, we assessed cell morphology and gene expression in LIF/serum conditions. Colony morphology was indistinguishable between the parental E2A-V5 cell line and *E2A*^−/−^ and *E2A*^−/−^*HEB*^−/−^ cells, and immunostaining showed similar levels of expression of the pluripotency markers Oct4 and Nanog between knockout and parental cells (Fig. 3A), indicating normal self-renewal. qRT-PCR analysis of pluripotency and lineage markers in LIF/serum further confirmed that there were no differences in expression levels between parental and knockout cell lines (Fig. 3B).

We next examined the ability of the knockout cells to differentiate under standard neural monolayer conditions. qRT-PCR analyses showed that, compared with controls, *E2A*^−/−^ and *E2A*^−/−^*HEB*^−/−^ cells were unable to robustly upregulate markers of neural differentiation, including *Sox1*, *Foxd4* and *Pax3* (Fig. 3B; Fig. S5). Furthermore, although knockout cells were able to downregulate expression of the naïve pluripotency marker *Nanog*, they maintained comparably high expression of the epiblast markers *Oct4* and *Fgf5*. Immunostaining of differentiation cultures for Sox1, Oct4 (Fig. 3C) and the marker of immature neurons, Tuj1 (Fig. 3D), further supported the gene expression data. Interestingly, we also observed that the disruption of E-protein expression resulted in an upregulation of the mesendodermal lineage markers, *T* (*brachyury*) and *Eomes* (Fig. 3B,D), as well as re-expression of *Nanog*, suggesting that these cells were beginning to adopt an identity more similar to the proximal epiblast, despite being cultured under neural differentiation conditions. Furthermore, we noted that both the neural differentiation defect and the upregulation of mesendodermal markers were more pronounced when HEB was deleted in addition to E2A, indicating that HEB is able to functionally compensate for E2A, to a limited extent, in this context. Taken together with the overexpression data, these findings suggest that E2A is both sufficient and required for efficient neural differentiation of ESCs.

### Identification of early E2A response genes

Having identified a novel role for E2A homodimers in driving the neural differentiation of ESCs, we next sought to identify the downstream targets of E2A in this process. We have previously reported that BMP blocks neural differentiation by maintaining E-cadherin (Malaguti et al., 2013), and others have reported that E2A is a direct transcriptional repressor of E-cadherin (Pérez-Moreno et al., 2001). Combined with our earlier observation that forced homodimer expression causes a downregulation of E-cadherin mRNA expression (Fig. 2B), we hypothesised that E2A homodimers may also repress E-cadherin protein expression to enable neural differentiation. However, contrary to this hypothesis, we detected no significant downregulation of E-cadherin protein in response to the induction of E2A homodimers before the expression of Sox1 (Fig. S6A). Our findings do not exclude the possibility that E-cadherin is a transcriptional target of E2A in pluripotent cells but they do suggest that E-cadherin is unlikely to mediate the effects of E2A on neural differentiation.

**Fig. 4.**
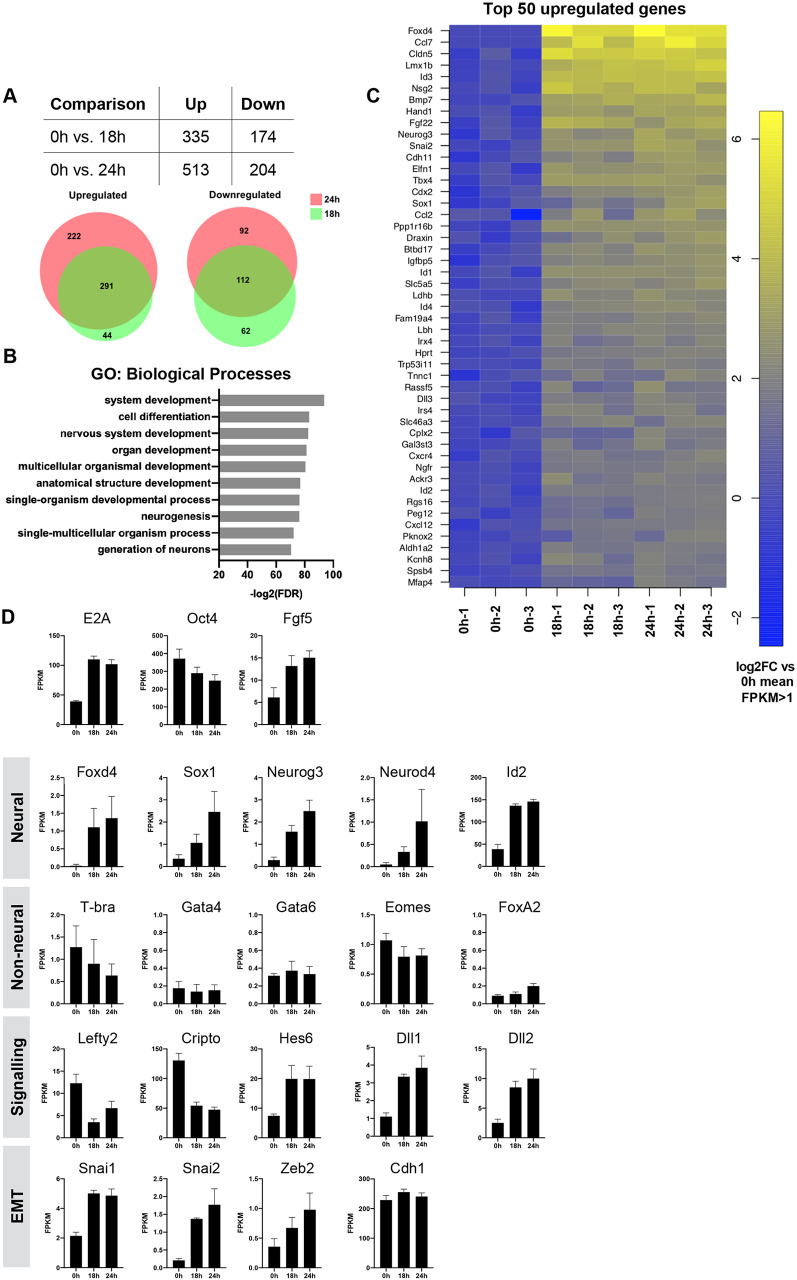
**Global transcriptome profiling by RNA-seq reveals E2A homodimers specifically upregulate neural lineage markers.** (A) Differentially expressed genes in each comparison according to a minimum threshold of log2 fold-change of 2 and a maximum FDR of 0.05. Proportional Venn diagrams illustrate the overlap of differentially expressed genes between timepoints. (B) GO analysis of the top 50 upregulated genes at 24 h. (C) Heatmap of the top 50 genes upregulated at 24 h. (D) Mean FPKM (fragments per kilobase per million reads mapped) RNA-seq expression values for selected genes at 0 h, 18 h and 24 h. Data are mean±s.d., *n*=3 biological replicates.

To identify novel downstream targets and examine genome-wide changes in expression in response to the induction of E2A homodimers, an RNA-seq approach was chosen. One aim of this approach was to identify factors that mediate the ability of E2A to upregulate Sox1. Expression of *Sox1* transcript was robustly upregulated by 18 h compared with the corresponding no dox control, whereas expression of Sox1 protein was first detected at 24 h (Fig. S6B,C). Based on these observations we performed RNA-seq analysis at 0 h (no dox), 18 h and 24 h timepoints in triplicate to capture changes in genes acting upstream of Sox1. We found that of the 335 genes that were upregulated at 18 h compared with 0 h, 291 remained significantly upregulated at 24 h, whereas 112 out of the 174 genes downregulated at 18 h remained downregulated at 24 h (Fig. 4A).

### E2A homodimers specifically promote neural lineage commitment

Gene ontology (GO) analysis of genes upregulated at 24 h revealed an enrichment for terms associated with neural differentiation, including ‘nervous system development’, ‘neurogenesis’ and ‘generation of neurons’ (Fig. 4B). Further interrogation of the RNA-seq data highlighted an upregulation of neural lineage-associated genes, including *Foxd4* [log2 fold-change (logFC) 5.7; false discovery rate (FDR) 0.0003], *Sox1* (logFC 2.8; FDR 0.003), *Neurog3* (logFC 3.1; FDR 7.18E-05) and *Neurod4* (logFC 4.4; FDR 0.02) (Fig. 4C,D). In contrast, early markers of non-neural lineages, such as *T*, *Eomes*, *Gata4*, *Gata6* and *Foxa2* were not upregulated, suggesting that E2A homodimers specifically drive neural lineage commitment in ESCs. We additionally observed an upregulation of EMT-related genes, including *Snai1*, *Snai2* and *Zeb2*, but no detectable early downregulation of E-cadherin, which was in line with our previous qRT-PCR analyses. We also found an upregulation of Notch signalling components, including *Dll1*, *Dll3* and the FGF target gene *Hes6* (Koyano-Nakagawa et al., 2000; Murai et al., 2007), and downregulation of Nodal pathway genes, including the Nodal co-receptor *Cripto* (*Tdgf1*) and the Nodal target gene *Lefty2*. Taken together, these data suggest that E2A homodimers are able to dominantly induce a transcriptional programme associated with the early stages of neural differentiation, although it remains possible that additional factors might be required to enable cells to progress through the later stages of neuronal differentiation.

### E2A drives neural commitment by upregulating Foxd4 and dampening Nodal signalling

The forkhead box TF Foxd4 is a likely candidate for mediating the effect of E2A on neural differentiation. Foxd4 was identified by RNA-seq as the most highly upregulated gene at 24 h following E2A homodimer induction (Fig. 4C), and is required for neural fate acquisition in mouse ESCs (Sherman et al., 2017). This is consistent with our observation that *Foxd4* is transiently upregulated at day 3 of neural differentiation, before overt *Sox1* expression, whereas E2A^−/−^ and *E2A*^−/−^*HEB*^−/−^ cells fail to upregulate *Foxd4* (Fig. 3B).

**Fig. 5.**
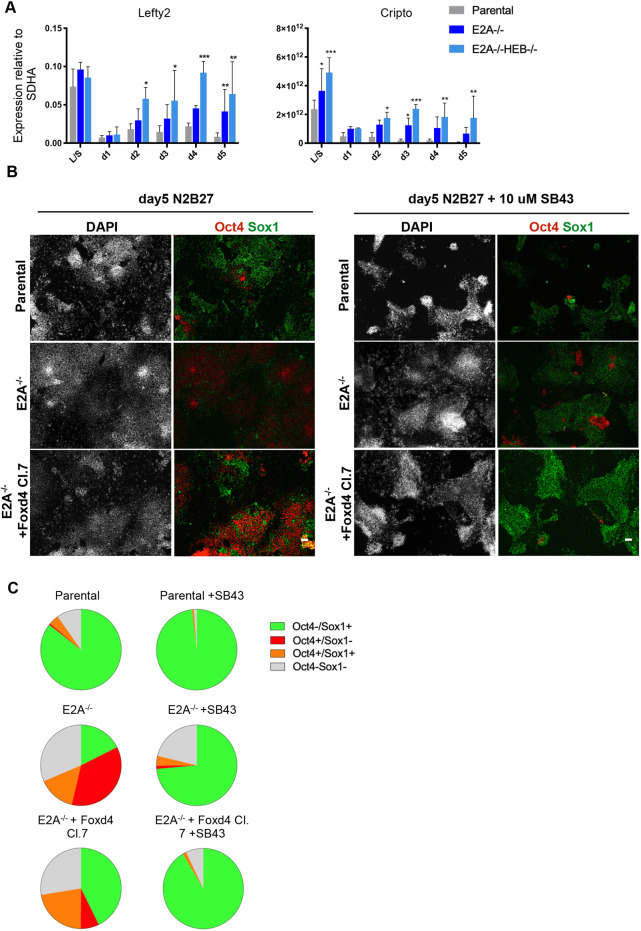
**Combined Foxd4 expression and Nodal signalling inhibition rescues differentiation defect in E-protein knockout cells.** (A) qRT-PCR analysis of Nodal pathway gene expression in knockout and parental ESC line differentiation. Data are mean±s.d., *n*=3 biological replicates. *P<0.05, **P<0.01 and ***P<0.001, two-tailed paired Student's *t*-test. (B) Immunostaining of parental and knockout cells at day 5 of differentiation ±10 µM SB43. Scale bars: 50 µm. (C) Quantification of immunostaining of cells at day 5 of differentiation ±10 µM SB43 performed by quantitative image analysis. Data are the mean values for three biological replicates. A minimum of 8000 nuclei were scored per experiment. Data for all replicates are shown in Table S3.

Alternatively, E2A could be modulating differentiation by dampening Nodal activity. The Nodal pathway genes *Cripto* and *Lefty2* were found to be downregulated in response to E2A induction (Fig. 4D). During gastrulation, the anterior epiblast fated for neuroectoderm is ‘silent’ for Nodal signalling (Peng et al., 2016), and inhibition of the pathway has been shown to promote neural differentiation in both mouse and human ESCs (Vallier et al., 2004; Watanabe et al., 2005; Camus et al., 2006). We found that although *Cripto* and *Lefty2* were rapidly downregulated and maintained at basal levels during the differentiation of control cells, they were progressively upregulated in E-protein knockout lines, with a more dramatic effect evident in *E2A*^−/−^*HEB*^−/−^ cells (Fig. 5A).

Based on these observations, we hypothesised that Foxd4 and/or a dampening of Nodal signalling could explain how E2A can drive neural differentiation. To test this, we sought to determine whether ectopic expression of Foxd4 or inhibition of Nodal signalling could rescue the neural differentiation defect observed in E-protein knockout cells.

We first generated *E2A^−/−^* ESC lines that ectopically express Foxd4. A plasmid encoding Foxd4 cloned upstream of an internal ribosomal entry site (IRES)-puromycin resistance cassette, under the control of a PGK promoter, was assembled and transfected into *E2A^−/−^* cells. *Foxd4* expression was assessed in undifferentiated ESCs by qRT-PCR (Fig. S7A) and three clonal lines were chosen for subsequent analyses (clones 3, 7 and 8). Ectopic expression of Foxd4 was not sufficient to force neural differentiation of *E2A^−/−^* ESCs cultured in LIF/serum, as assessed by *Sox1* expression (Fig. S7A). However, using quantitative immunostaining analysis, we observed that when cells were placed under differentiation conditions, ectopic expression of Foxd4 was able to partially restore *Sox1* expression in knockout cells (Fig. 5B,C; Fig. S7B; Table S3) and to promote a large proliferation of the Oct4-expressing cells present in the culture (Fig. 5B,C).

We then sought to test whether inhibition of the Nodal pathway using SB431542 (SB43), which inhibits the activity of TGF-β receptors (Inman et al., 2002), could restore neural differentiation in E-protein knockout cells. We observed that SB43 treatment increased the proportion of Sox1 expression in control cells, and resulted in a strong upregulation of Sox1 in *E2A*^−/−^ and *E2A*^−/−^*HEB*^−/−^ lines. When applied to cells also ectopically expressing Foxd4, SB43 was able to effect a robust differentiation response (Fig. 5B,C; Fig. S7B,C). Taken together, these results suggest an additive effect of ectopic Foxd4 expression and TGF-β pathway inhibition in restoring the differentiation capacity of *E2A*^−/−^ cells. We conclude that E2A homodimers promote neural differentiation by upregulating Foxd4 while dampening the activity of TGFβ ligands.

## DISCUSSION

The key extracellular signalling pathways that inhibit differentiation of ESCs are now well established (Haegele et al., 2003; Smith et al., 2008; Ying et al., 2003a; Zhang et al., 2010). A considerable amount of progress has also been made in elucidating the network of TFs that control later aspects of mammalian neuronal specification and differentiation (Imayoshi and Kageyama, 2014). Much less is known, however, about the molecular processes that link these two events at the earliest stages of neural differentiation. Recent work has identified roles for TFs such as Zfp521, Oct6 and Zic1/2 as intrinsic regulators of early neural differentiation (Kamiya et al., 2011; Zhu et al., 2014; Sankar et al., 2016). However, although it has been demonstrated that Oct6 is involved in the activation of several neural fate-promoting genes (Zhu et al., 2014), Zfp521 and Zic1/2 are more likely playing a role in consolidating, rather than initiating, neural fate (Kamiya et al., 2011; Iwafuchi-Doi et al., 2012). In this study, we sought to identify novel intrinsic regulators of neural fate commitment in ESCs, and to elucidate the molecular events unfolding downstream of the BMP signalling pathway during this process.

The HLH transcription factor Id1 has previously been identified as a key effector of the BMP pathway, and its overexpression has been shown to block entry into the neural lineage and promote differentiation to alternative fates (Ying et al., 2003a,b; Malaguti et al., 2013). Although the function of Id proteins as dominant-negative inhibitors of bHLH transcription factors is well characterised in heterologous systems (Norton, 2000), it has not previously been investigated whether Id1 could also be functioning via this classical mechanism during neural commitment. Taken together with the observation that E2A is the major binding partner of Id1 in ESCs (Davies et al., 2013), we therefore hypothesised that Id1 maintains pluripotency by sequestering E2A, thus preventing it from forming functional homodimers or heterodimers that could otherwise initiate a differentiation response.

Despite their widespread expression during embryogenesis (Pérez-Moreno et al., 2001), a definitive role for Class I bHLH TFs in early neural development has not been explored, with E-proteins often relegated to simply being obligate dimerisation partners for tissue-specific Class II bHLH factors. Single E-protein knockouts in mice have also failed to uncover any robust neurodevelopmental deficiencies, and attempts to generate E-protein compound knockouts to overcome the expected problem of functional compensation between family members have thus far been unsuccessful as even mice with only single E-proteins knocked out do not survive beyond 2 weeks after birth (Ravanpay and Olson, 2008). In *Drosophila*, however, loss of the only Class I bHLH factor gene, *daughterless*, has been found to result in neural differentiation defects (Caudy et al., 1988).

In this study we explored a role for E2A in neural fate commitment. Generation of an endogenously tagged E2A-V5 ESC line enabled us to define the expression of E2A from pluripotency to the onset of differentiation on the single cell level. Using a doxycycline-inducible system, we report that overexpression of E2A is sufficient to promote commitment to the neural lineage in ESCs, even under non-permissive culture conditions. Specifically, we found that forced E2A homodimers, rather than E2A-bHLH heterodimers, appear to drive this process, as the introduction of a single amino acid change (C570A) into E2A to disrupt homodimer stability, while still maintaining heterodimerisation capacity (Benezra, 1994), did ablate the ability of the forced homodimer to promote *Sox1* expression. These findings suggest that Id proteins inhibit neural differentiation, at least in part, by suppressing homodimerisation of E proteins: this would to our knowledge be the first example of a physiological role for E2A homodimers outside of the context of B-cell specification (Shen and Kadesch, 1995). It would be interesting to test this further by investigating whether E2A forced homodimers render neural differentiation resistant to the anti-neural effects of Id proteins, and whether introducing a C570A mutation into endogenous E2A recapitulates the neural defects seen in E2A null cells.

Interestingly, we also found that overexpression of E2A was able to drive upregulation of its own negative regulator, *Id1*, consistent with previous descriptions of a Class I/Class V HLH feedback loop in *Drosophila*, in which overexpression of the E-protein *daughterless* causes transcriptional upregulation of the Id protein Extra macrochaetae (Bhattacharya and Baker, 2011). We recognise that our forced homodimerisation of E2A is likely to disrupt this negative-feedback loop, and that this effect might help amplify the pro-neural activity of E2A homodimers.

The findings from the gain-of-function assays were further supported by data from the loss-of-function studies. Generation of novel *E2A*^−/−^ and *E2A*^−/−^*HEB*^−/−^ ESC lines highlighted that although knockout cells were able to self-renew normally, they were not able to differentiate efficiently. Additionally, although knockout cells were able to downregulate *Nanog*, they maintained relatively high expression of the epiblast markers *Fgf5* and *Oct4*. This suggests that although knockout cells are able to navigate the exit from pluripotency efficiently, they fail to complete the proposed second stage of neural lineage commitment involving the transition from epiblast-like cells to neuroectoderm-like cells (Zhang et al., 2010). The observation that *E2A*^−/−^*HEB*^−/−^ cells displayed a more pronounced neural differentiation defect suggests that HEB is, indeed, able to at least partially compensate for the loss of E2A in this context. This is in line with previous studies that have demonstrated that HEB is able to functionally replace E2A during B-cell commitment (Zhuang et al., 1998), highlighting the extent of redundancy between E-protein family members. That *E2A*^−/−^*HEB*^−/−^ cells also appear to preferentially upregulate markers of mesendodermal lineages, including *T* and *Eomes*, is a potentially interesting topic of investigation for future studies, especially considering that HEB (and E2A) have previously been implicated in promoting mesendodermal lineage specification, both in *Xenopus* and in human ESCs (Yoon et al., 2011; Li et al., 2017).

In this study, we identified *Foxd4* as a key gene strongly upregulated in response to forced E2A homodimers, positioning it upstream of Sox1 expression during neural commitment. These data are in line with recent observations also made in ESCs (Sherman et al., 2017), and with the reported expression of *Foxd4* in the mouse neuroectoderm at E7.5 (Kaestner et al., 1995). The *Xenopus* homologue of Foxd4, Foxd4l1 (previously Foxd5), has a well-established role as part of a broader network of neural fate stabilising factors (Yan and Moody, 2009), and it has been shown to expand the population of progenitor cells in the immature neuroectoderm, while repressing the transcription of genes associated with neural differentiation (Sullivan et al., 2001). A potential role for Foxd4 in maintaining neuroectodermal cells in a proliferative non-differentiating state, therefore, might also explain the large proliferation of Oct4^+^ cells we observed when Foxd4 was ectopically expressed in E2A^−/−^ cells.

We also reported that components of the Nodal signalling pathway were downregulated upon the overexpression of E2A homodimers, and conversely upregulated during the differentiation of E-protein knockout cells. Interestingly, E2A has also been shown to repress the transcription of *lefty* in *Xenopus*, with knockout of E2A causing upregulation of *lefty* and a subsequent failure in mesendodermal fate specification (Wills and Baker, 2015). We propose that E2A could be playing a similar role to repress Nodal signalling in mouse pluripotent cells, but during neural fate commitment – a process in which the inhibition of Nodal signalling is already known to be important in both mouse and human pluripotent cells (Watanabe et al., 2005; Vallier et al., 2009). Given that the inhibition of Nodal also promotes neural differentiation of human pluripotent cells (Vallier et al., 2004; Chambers et al., 2009) and that E2A regulates lefty in human cells (Yoon et al., 2011), it seems likely that E2A might also drive neural differentiation of human pluripotent cells, although this remains to be tested. It is likely that E2A has multiple downstream effectors in mammalian cells, as it does in *Xenopus* (Wills and Baker, 2015), but our rescue experiments suggest that the inhibition of Nodal and the activation of Foxd4 are key effectors that explain the ability of E2A to drive neural differentiation. In summary, we propose that E2A plays an instructive, rather than passive, role in promoting neural fate commitment in pluripotent cells by promoting the transcription of neural lineage genes while simultaneously suppressing the Nodal signalling pathway.

## MATERIALS AND METHODS

### Mouse ESC culture and neural differentiation

E14tg2α mouse ESCs were used as wild-type cells. All cell lines used in this study were screened for mycoplasma contamination. ESCs were maintained in Glasgow minimum essential medium supplemented with β-mercaptoethanol, non-essential amino acids, glutamine, pyruvate, 10% fetal calf serum (FCS) and 100 units/ml LIF on gelatinised tissue culture flasks (Smith, 1991). Monolayer neural differentiation was performed as described by Pollard et al. (2006). Briefly, ESCs were washed in Dulbecco's modified eagle medium (DMEM)/F12 to remove all traces of serum and plated at 1×10^4^ cells/cm^2^ in N2B27 medium on gelatinised tissue culture plates. N2B27 consists of a 1:1 ratio of DMEM/F12 and Neurobasal media supplemented with 0.5% N2, 0.5% B27, L-glutamine and β-mercaptoethanol. For Nodal signalling inhibition experiments, cells were seeded in N2B27 supplemented with 10 µM SB431542.

### CRISPR/Cas9 epitope tagging of ESC lines

Guide RNA was manually designed to introduce the V5 tag into the 3′ UTR of E2A based on the annotation of the final coding exon and the 3′ UTR sequence. A ∼200 bp sequence around the stop codon was used as an input for guide RNA design using either crispr.mit.edu or crispor.tefor.net web-based design tools. High-scoring guide RNAs were chosen based on minimal predicted off-target cleavage events, and having a cut site within the 3′ UTR, preferably within 10 bp of the stop codon. For ssODN design, the V5 tag sequence was flanked by ∼75 bp homology arms and a PAM-blocking mutation (NGG>NGC) was introduced into the 3′ UTR sequence to prevent re-cutting of donor DNA by Cas9. Ribonucleoprotein (RNP) complexes were assembled as described by Dewari et al. (2018). Briefly, crRNA and tracrRNA oligos were mixed in equimolar concentration, heated at 95°C for 5 min and allowed to cool to room temperature to anneal. Recombinant Cas9 protein (10 μg) was added to the annealed cr/tracrRNAs to form the RNP complex, which was incubated at room temperature for 20 min and stored on ice until electroporation. ssODN (30 pmol) was added to the RNP complex immediately before electroporation. ESCs (5×10^4^) were transfected using the 4D Amaxa nucleofection system (Lonza) using the optimised program CA-210. Following transfection, cells were transferred into a six-well plate and allowed to recover for 3 to 5 days in ESC media. Clonal cell lines were isolated from bulk populations by manual colony picking and were subsequently analysed for successful knock-in by PCR genotyping, immunocytochemistry and Sanger sequencing of a ∼500 bp region spanning the target site.

Synthetic Alt-R crRNA, tracrRNA, ssODN Ultramer template DNA oligonucleotides and recombinant Cas9 protein were manufactured by Integrated DNA Technologies. Guide RNA and ssODN DNA sequences are provided in Table S1.

### Knockout ESC line generation

For generation of *E2A*^−/−^ ESCs, guide RNAs were designed to target exon 3 of the E2A gene. Targeting was performed on the E2A-V5 cell line to facilitate knockout line validation by loss of V5 signal. For generation of *E2A*^−/−^*HEB*^−/−^ double knockout ESCs, exon 9 of the *HEB* locus was targeted in E2A^−/−^ knockout cells (clone 9) in order to disrupt both HEBcan and HEBalt splice variants. Guides were designed, assembled as RNPs with recombinant Cas9 and transfected as detailed above. Knockouts were verified by Sanger sequencing of targeted allele and western blot analysis using mouse anti-V5 (1:1000; Thermo Scientific, R960-25) and mouse anti-HEB (1:200; Santa Cruz Biotechnology, sc-28364) antibodies. Experiments presented in the main text were performed using *E2A*^−/−^ clone 9 and *E2A*^−/−^*HEB*^−/−^ clone 12.

### Doxycycline-inducible E2A cell lines

The E47 monomer construct comprises an N-terminally FLAG-tagged mouse E47 sequence. To generate the E47 forced homodimer construct, the DNA sequence corresponding to the FLAG-tagged mouse E47 was tethered at its C-terminus to the N-terminus of a second mouse E47 sequence with a 13-amino acid flexible linker sequence of TGSTGSKTGSTGS by overlapping extension PCR (Malaguti et al., 2019). For generation of the C570A homodimerisation-deficient cell lines, mutations were introduced into both E47 sequences in the forced dimer construct using PCR site-directed mutagenesis and verified by Sanger sequencing. Inducible cells lines were generated using an inducible cassette exchange technique (Iacovino et al., 2013). Cells were stimulated with 1 µg/ml doxycycline in all experiments.

### Generation of Foxd4 rescue lines

The full-length *Foxd4*cDNA sequence was cloned into a pPGK-IRES-puromycin resistance plasmid. *E2A*^−/−^ (clone 9) cells were transfected with the resulting construct using the 4D Amaxa nucleofection device (Lonza) and clonal lines were isolated by puromycin selection (1 μg/ml). Following clonal line expansion, ESCs were characterised by qRT-PCR for *Foxd4* expression.

### Immunocytochemistry, western blot analysis and flow cytometry

For immunocytochemistry, samples were fixed with 4% paraformaldehyde for 10 min at room temperature and incubated with blocking buffer (PBS, 0.1% Triton X-100, 3% donkey serum) for 30 min at room temperature. Primary antibodies were diluted in blocking buffer and incubated with cells overnight at 4°C. After three washes with PBS, cells were incubated with secondary antibodies conjugated with Alexa fluorophores (Life Technologies) diluted 1:1000 in blocking buffer for 1 h at room temperature. For nuclear counterstaining, cells were incubated with 1 µg/ml DAPI (Sigma-Aldrich) for 5 min following immunostaining. Cells were washed a minimum of three times in PBS before imaging. For confocal imaging, cells were plated onto glass coverslips, stained and mounted in ProLong Gold Antifade Reagent (Life Technologies). Cells were either imaged on the Leica SP8 confocal microscope or the Olympus IX-81 widefield microscope. All images were analysed in Fiji. Primary antibodies used for immunocytochemistry were: mouse anti-β-III-tubulin/Tuj1 (1:1000; BioLegend, MMS-435P), rat anti-Nanog (1:200; Thermo Fisher Scientific, 14-5761-80), goat anti-Oct4 (1:200; Santa Cruz, sc-8628), mouse anti-Sox1 (1:200; BD Pharmingen, 560749), rabbit anti-Sox2 (1:200; Abcam, ab97959) and mouse anti-V5 (1:200-500; Thermo Fisher Scientific, R960-25). Secondary antibodies used in immunostaining experiments were: donkey anti-mouse Alexa Fluor 488 (Thermo Scientific, A21202), donkey anti-mouse Alexa Fluor 568 (Thermo Scientific, A10037), donkey anti-rabbit Alexa Fluor 488 (Thermo Scientific, A21206), donkey anti-rabbit 568 (Thermo Scientific, A10042), donkey anti-rat Alexa Fluor 488 (Thermo Scientific, A21208), donkey anti-rat Alexa Fluor 594 (Thermo Scientific, A21209) and donkey anti-goat Alexa Fluor 647 (Thermo Scientific, A21447).

For western blot analysis, cells were lysed in radioimmunoprecipitation assay buffer+1× PMSF (Alpha Diagnostics). Protein lysate (20 μg) was run on 4%-12% NuPAGE Bis-Tris Gel (Novex) and transferred onto Amersham Hybond ECL Nitrocellulose Membrane (GE Healthcare). Membranes were blocked in 5% Amersham ECL Prime Blocking Agent (GE Healthcare)+0.1% Tween 20 (Sigma-Aldrich) in PBS for a minimum of 1 h at room temperature. Membranes were incubated with primary antibody overnight at 4°C, washed in PBS+0.1% Tween 20, incubated in horseradish peroxide (HRP)-conjugated secondary antibody for 1 h at room temperature and washed in PBS+0.1% Tween 20. Membranes were subsequently incubated in Amersham ECL Western Blotting Detection Reagent (GE Healthcare) or Amersham ECL Prime Western Blotting Detection Reagent (GE Healthcare). Membranes were either used to expose Amersham Hyperfilm ECL (GE Healthcare) and developed using a Konica SRX-101A Medical Film Processor, or imaged using the Bio-Rad ChemiDoc system. Primary antibodies used in the western blot analysis were mouse anti-β-III-tubulin/Tuj1 (1:1000; Sigma-Aldrich, T5293), mouse anti-E2A (1:200; Santa Cruz Biotechnology, sc-416,), mouse anti-FLAG (1:5000; Sigma-Aldrich, F9291), mouse anti-HEB (1:200; Santa Cruz Biotechnology, sc-28364,), mouse anti-Sox1 (1:1000; BD Pharmingen, 560749) and V5 (1:500-1:1000; Thermo Fisher Scientific, R960-25). Secondary antibodies used were Amersham ECL Mouse IgG, HRP linked (GE Healthcare, NA931V) and Amersham ECL Rabbit IgG, HRP linked (GE Healthcare, NA934).

For flow cytometry analysis, cells were dissociated into a single cell suspension in PBS+10% FCS. Cells were stained with rat anti-E-cadherin (CD324), eFluor660-conjugated DECMA-1 (1:300; eBioscience, 50-3249-82) and 100 ng/ml DAPI to stain dead cells. Flow cytometry was performed on the BD Accuri C6 and analysis was performed using FlowJo software.

### Quantification of immunostaining

Immunofluorescence was quantified by nuclear segmentation based on DAPI signal and manual editing of segmentation results using Nessys software, as detailed by Blin et al. (2019).

### qRT-PCR

Total RNA was isolated from cells using the Absolutely RNA Miniprep Kit (Agilent). cDNA was generated using Moloney murine leukaemia virus reverse transcriptase and random primers. Primers and UPL probes (Roche) used are detailed in Table S2. All gene expression values were normalised to the housekeeping gene *Sdha.*

### RNA-seq

RNA was isolated from cells using the Absolutely RNA Miniprep Kit (Agilent) and RNA quality was verified using an Agilent 2100 Bioanalyzer. Subsequent cDNA library preparation, sequencing and bioinformatics analysis, including differential gene expression analyses, were performed by the Edinburgh Genomics facility. Library preparation was performed using the TruSeq Stranded mRNA Library Prep Kit (Illumina) and libraries were sequenced on the Illumina HiSeq4000. Reads were mapped to the mouse genome GRCm38 from Ensembl and were aligned to the reference genome using STAR (version 2.5.2b). Differential gene analysis was carried out with edgeR (version 3.16.5). Differentially expressed genes were assigned based on a minimum fold-change of two and a maximum FDR of 0.05. Gene ontology analysis was performed on genes upregulated at 24 h using the STRING database (Szklarczyk et al., 2017).

### Statistical analyses

qRT-PCR data are represented as mean±s.d. for a minimum of three experimental replicates. Statistical significance was calculated using Student's *t*-tests for pairwise comparisons, or ANOVA with correction for multiple comparisons for two or more samples. Statistically significant differences are shown as follows: *P<0.05, **P<0.01 and ***P<0.001.

## Supplementary Material

Supplementary information

Reviewer comments
