# Supplementary material for: The transcription factor E2A drives neural differentiation in pluripotent cells
Source: Development. 2020 Jun 22;147(12):dev184093. doi: 10.1242/dev.184093 (PMC7328008; doi:10.1242/dev.184093)
Supplement: Supplementary information [file develop-147-184093-s1.pdf]

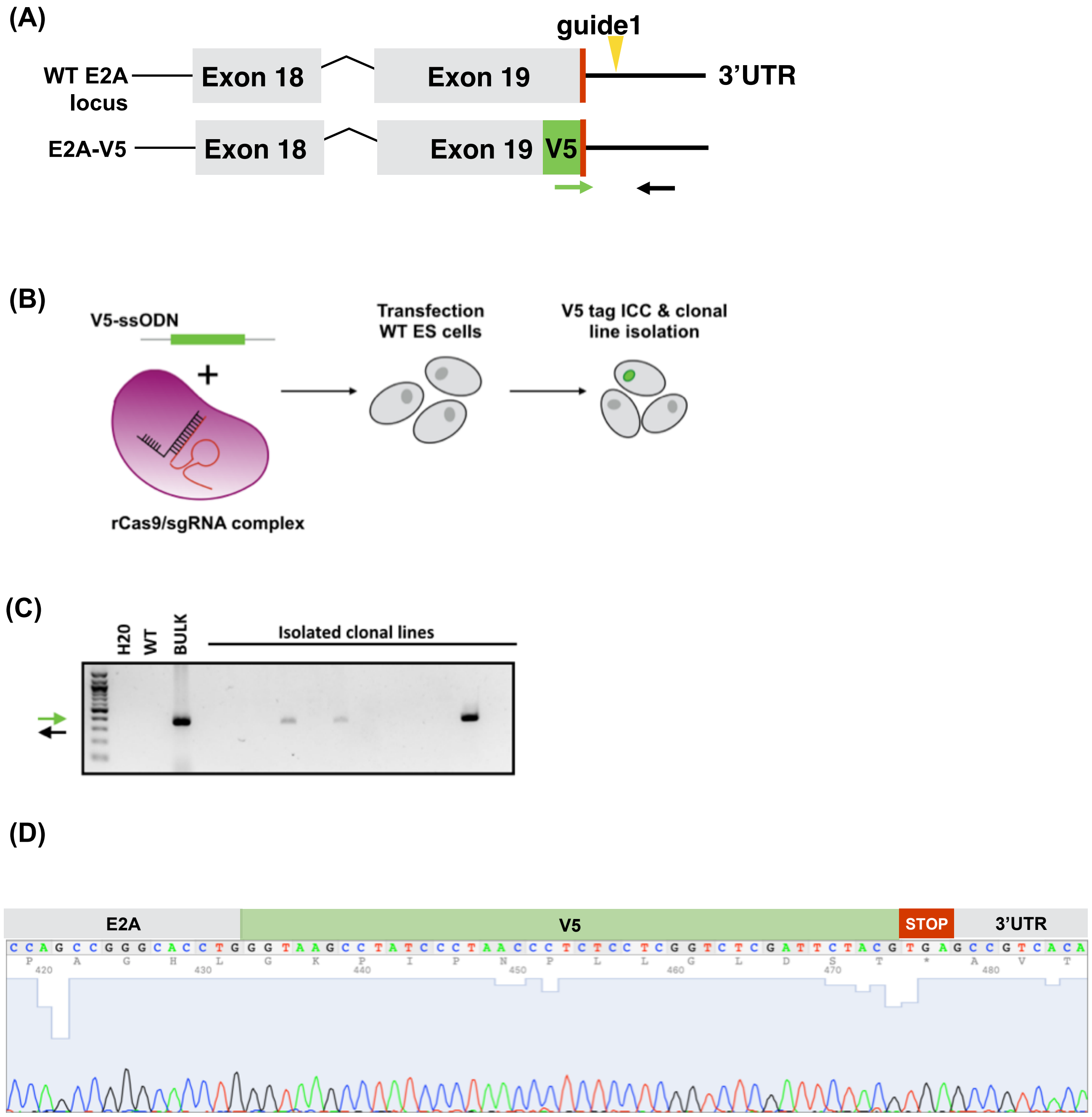

Figure S1. Generation and validation of endogenously tagged E2A-V5 ES cell lines.
(A) Schematic of exon targeting of E2A to generate tagged cell lines. V5 tag is shown in green, stop codon in red, and yellow arrow indicates location of the guide RNA (sgRNA) sequence.
(B) Method used to knock in the V5 tag by nucleofection of a complexed ribonucleoprotein (RNP) comprised of recombinant Cas9 protein (rCas9) and guide RNA into wild-type ES cells.
(C) PCR genotyping of derived clonal lines using primer pairs indicated by green and black arrows depicted in (A). Bands of correct size in clonal lines shown, compared to blank (H20), parental WT line, and bulk transfected population.
(D) Sanger sequencing trace of correctly targeted E2A-V5 clonal lines, confirming correct in-frame insertion of the V5 tag.

Development • Supplementary information

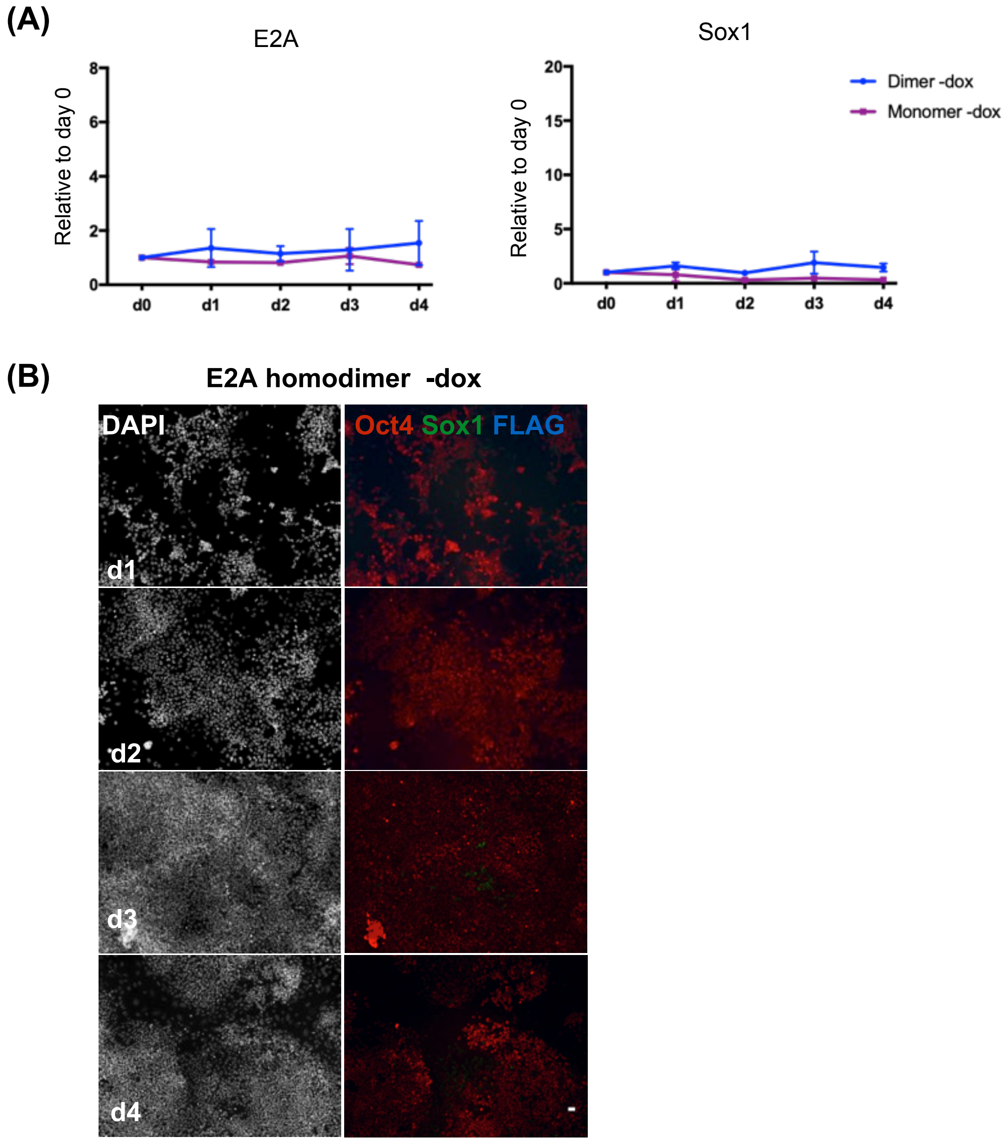

Figure S2. Sox1 is not expressed when inducible cells are cultured in LIF/serum without dox induction.

(A) qRT-PCR analysis of dox-inducible E2A monomer and forced homodimer cells cultured in LIF/serum without dox. Expression values are normalised to day 0. Error bars represent mean +/-SD of 2 biological replicates.

(B) Immunocytochemistry of inducible forced homodimer cells cultured in LIF/serum without dox. Scale bar: 30 μm

Development • Supplementary information

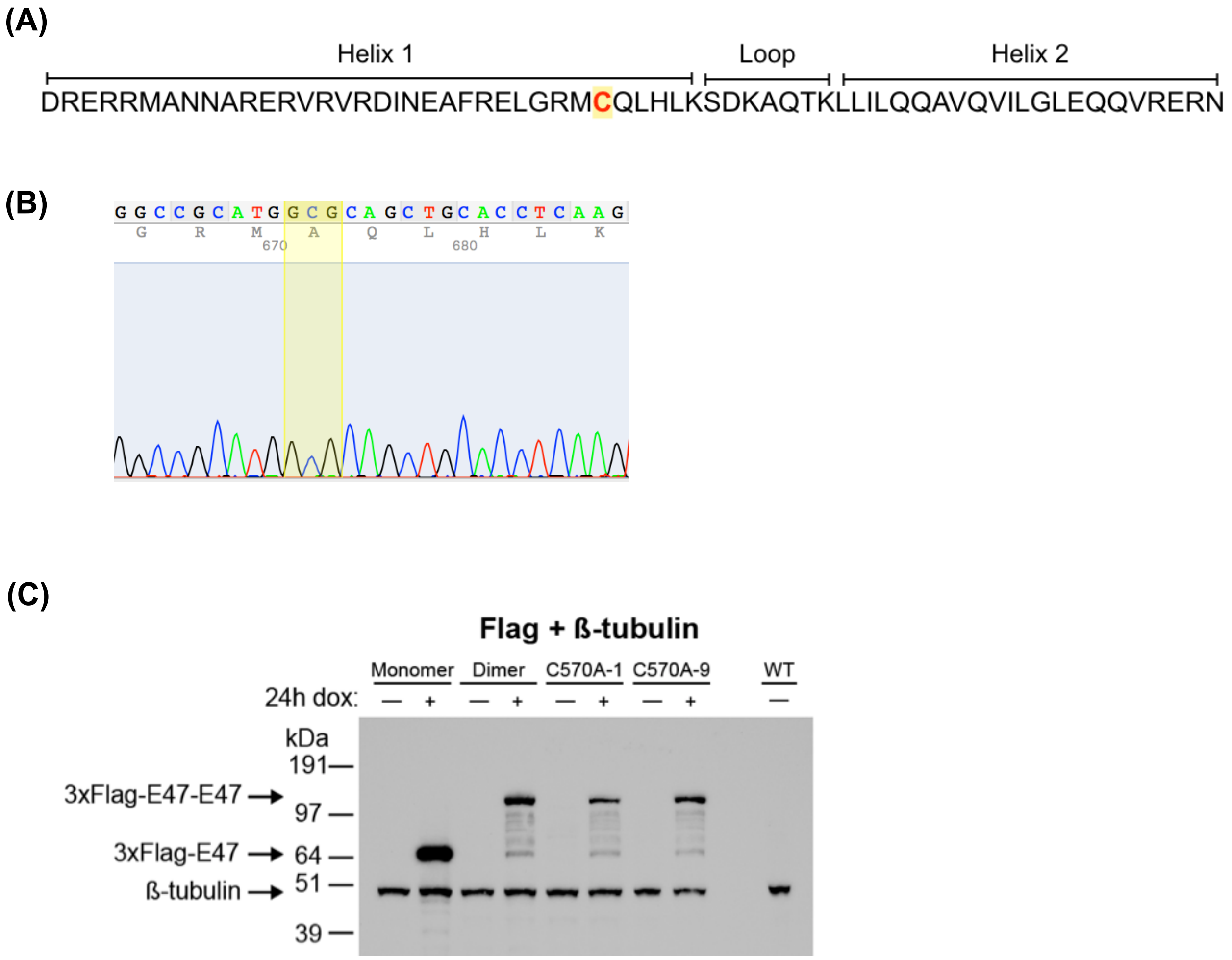

Figure S3. Wild-type and mutant forced homodimer cells.
(A) Sequence of the helix-loop-helix (HLH) domain of wild-type E2A with Cysteine-570 residue in helix 1 highlighted in yellow.
(B) Sanger sequencing trace of Cys>Ala (C570A) mutation introduced into both E2A monomer sequences in the forced homodimer construct to generate mutant forced homodimer inducible lines (C570A-1 and C570A-9).
(C) Western blot analysis of wild type monomer, forced homodimer and mutant forced homodimer cell lines +/- 24h of doxycycline induction. Membrane was blotted using anti-FLAG to detect the transgene and B-tubulin was used as a loading control.

Development • Supplementary information

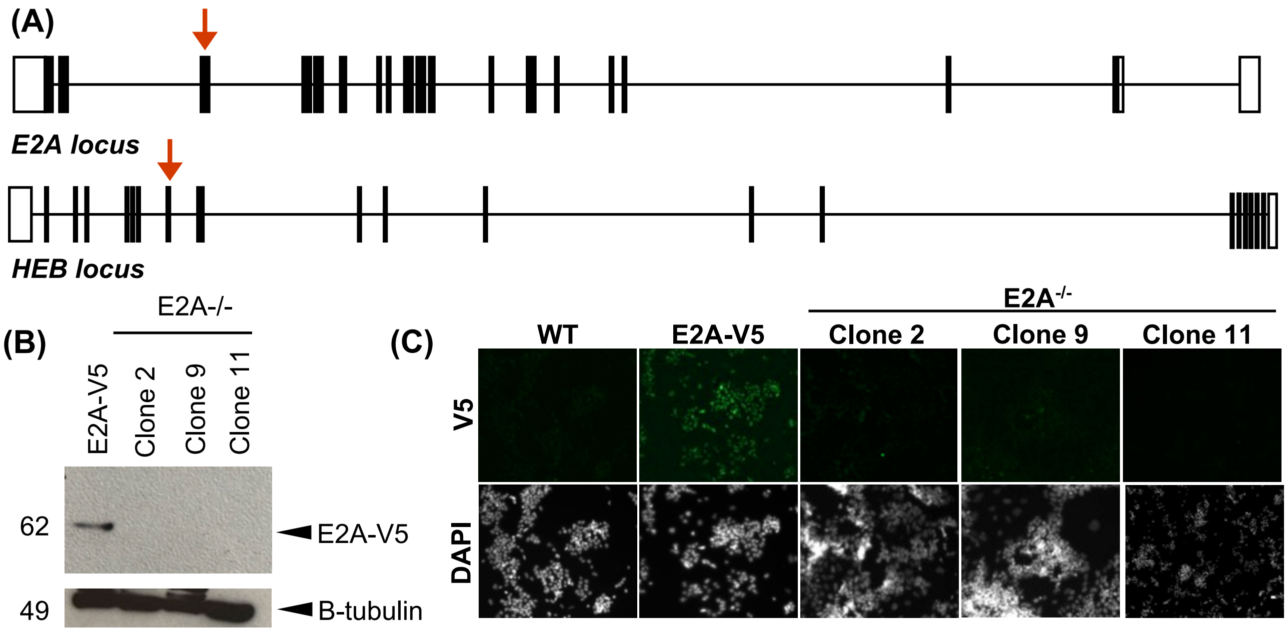

(D)

WT sequence	MFPLPVANGKSRPASLGGTQFAGS
ssODN sequence:	MFPLPVANGKSRPASLG*EFGTQFAGS
$E2A^{-/-}$ Cl. 2 allele 1&2	MFPLPVANGKSRPASLG*EFGTQFAGS
$E2A^{-/-}$ Cl. 9 allele 1	MFPLPVANGKSRPRLARVRIRNPVCRLR*D*
$E2A^{-/-}$ Cl. 9 allele 2:	MFPLPVANGKSRPASLHALSAPTVNPGLAS
$E2A^{-/-}$ Cl. 11 allele 1:	MFPLPVANGKSRPASPEPSLQAQVGLRGS
$E2A^{-/-}$ Cl. 11 allele 2:	MFPLPVANGKSRPASLG*EFGTQFAGS

(E)

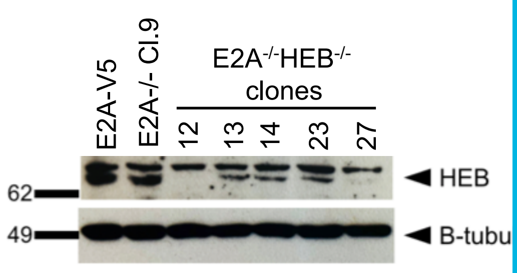

(F)

WT sequence	VYAPSPNSDDFNRESPSYPSPKPPTSMFASTFFMQ
ssODN sequence:	VYAPSANSD*EFLKYPYDVPDYADFNRESPSYPSPKPATSMFASTFFMQ
$E2A^{-/-}HEB^{-/-}$ Cl.12 allele 1	VYAPSANSD*EFLKYPYDVPDYADFNRESPSYPSPKPATSMFASTFFMQ
$E2A^{-/-}HEB^{-/-}$ Cl.12 allele 2	VYAPSANSD*EFLKYPYDVPDYADFNRESPSYPSPKPATSMFASTFFMQ
$E2A^{-/-}HEB^{-/-}$ Cl. 27 allele 1	VYAPS - - - - - - - - RESPSYPSPKPPTSMFASTFFMQ
$E2A^{-/-}HEB^{-/-}$ Cl. 27 allele 2	VYAPSPNSD*EFLKYPTMFQITLISTVNLLVTHLP

Figure S4. Validation of E-protein knockout lines

(**A**) Structure of *E2A* and *HEB* loci. Red arrow indicates Cas9/sgRNA target site. (**B**) Western blot of E2AV5 Cl.74 (parental cell line) and three E2A knockout clones 2, 9 & 11 using anti-V5 antibody. E2A-V5=69kDa. B-tubulin was analysed as a loading control. (**C**) Immunostaining of WT, E2A-V5 (parental) and $E2A^{-/-}$ clonal lines with anti-V5 antibody. Scale bar: 30 µm (**D**) Sanger sequencing of $E2A^{-/-}$ clones 2, 9 and 11. WT exon 3 and ssODN template DNA amino acid sequences shown with mutations highlighted in red. $E2A^{-/-}$ clone 2 has biallelic knock-in of the stop codon-containing ssODN template sequence, clone 9 has two independently disrupted E2A alleles and clone 11 has heterozygous knock-in of the ssODN and a second non-homologous end joining (NHEJ)-disrupted allele. (**E**) Western blot of E2AV5 Cl74, $E2A^{-/-}$ clone 9 (parental) and $E2A^{-/-}HEB^{-/-}$ clonal lines using anti-HEB antibody. HEB=85kDa. B-tubulin was analysed as a loading control. (**F**) Sanger sequencing of $E2A^{-/-}HEB^{-/-}$ clones 12 and 27. Targeted WT exon 9 and ssODN template DNA amino acid sequences shown above, with mutations highlighted in red. $E2A^{-/-}HEB^{-/-}$ clone 12 has biallelic knock-in of the ssODN sequence, and clone 27 has two independently disrupted HEB alleles; one allele contains an 8 amino acid deletion, the other allele contains a premature stop codon.

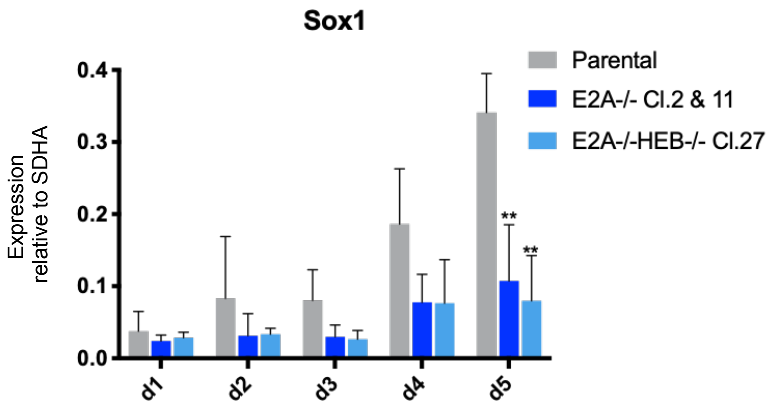

Figure S5. Transcriptional analysis of additional E-protein knockout clones. qRT-PCR analysis of two additional E2A$^{-/-}$ clones (clones 2 and 11) and one additional E2A$^{-/-}$HEB$^{-/-}$ clone (clone 27) differentiated in N2B27. Relative expression values shown are the mean of three independent experiments for each clonal line.

Development • Supplementary information

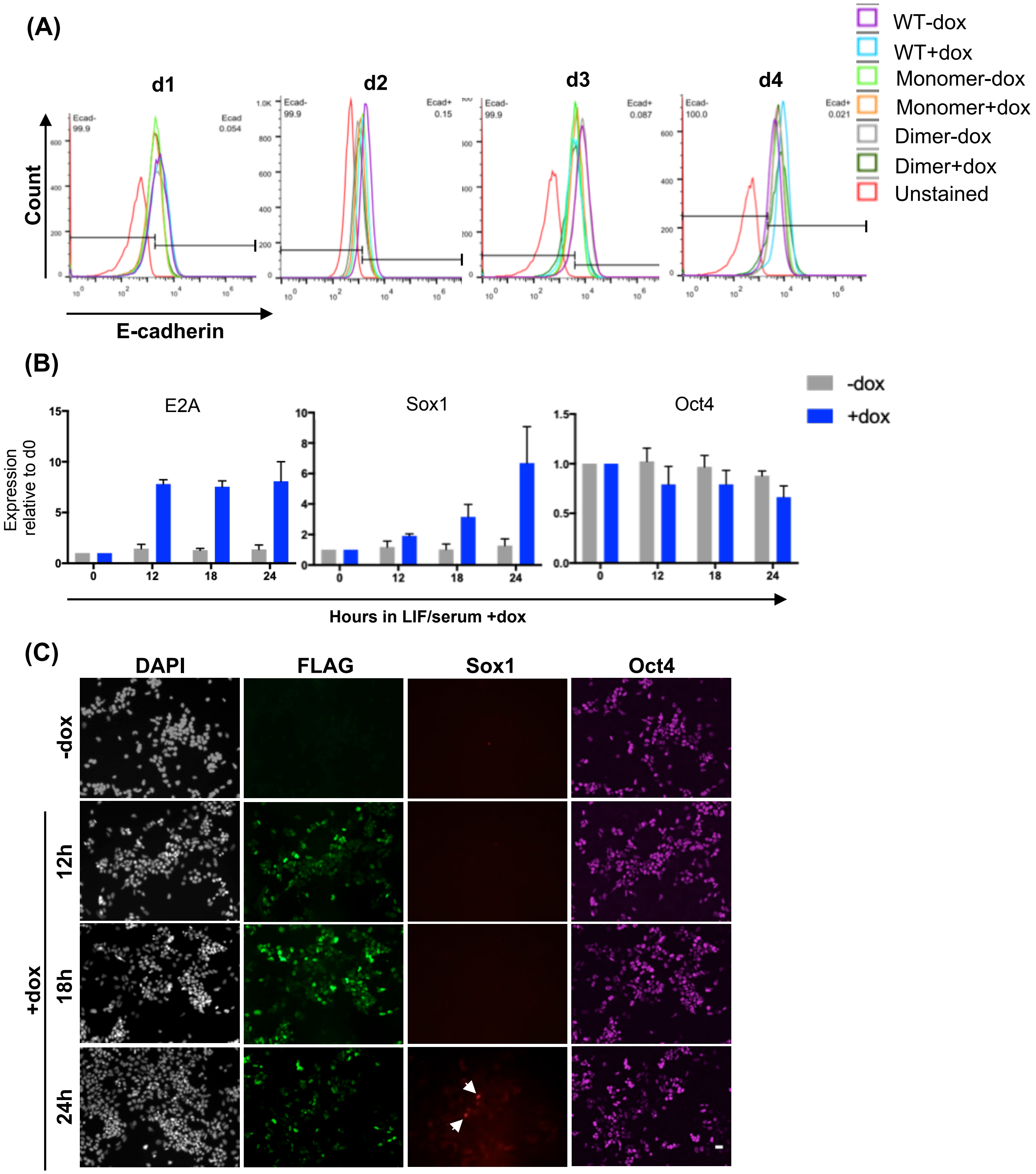

Figure S6. Identifying a time frame for detection of early E2A homodimer response genes.
(**A**) Flow cytometric analysis of the effect of overexpression of E2A monomers and homodimers on E-cadherin (E-cad) protein expression. WT ES cells are included as a control and were used in combination with unstained WT cells to define gates. Experiments were repeated twice and the data from replicates follow the same trend as displayed in the figure. (**B**) qRT-PCR analysis of cells following 12, 18 and 24 hours of E2A homodimer induction in LIF/serum. No dox controls are also shown. Error bars represent standard deviation of three biological experiments, which were used for subsequent RNA-sequencing analysis. (**C**) Immunostaining of cells during the 24h timecourse to assess transgene activation using an anti-FLAG antibody, and co-staining with anti-Sox1 and anti-Oct4. White arrows highlight small number of cells that express Sox1 protein 24 hours post-induction. Scale bar: 50 μm

Development • Supplementary information

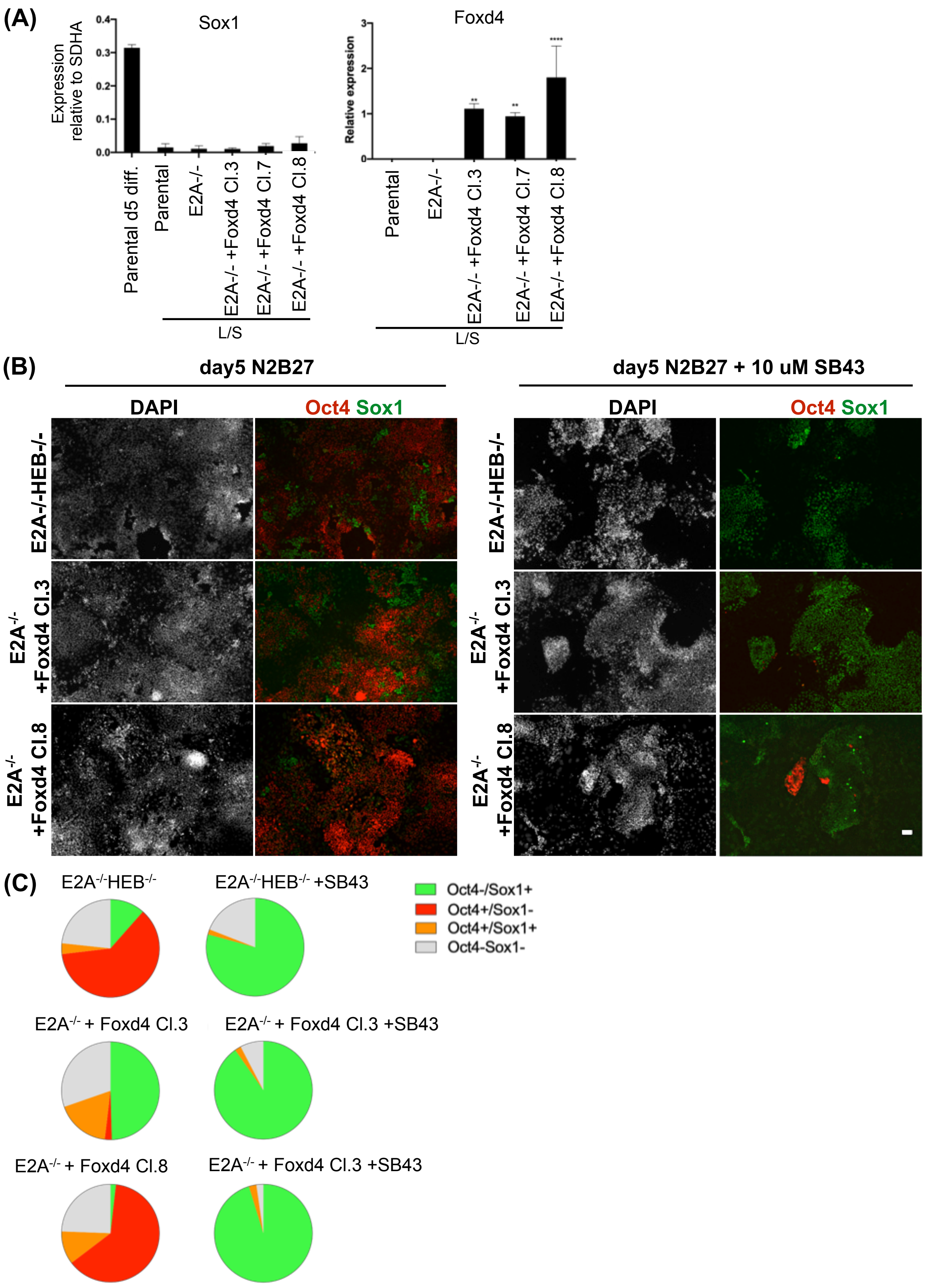

Figure S7. Analysis of additional clonal lines ectopically overexpressing Foxd4 in an E2A$^{-/-}$ background.

(**A**) qRT-PCR analysis of knockout and Foxd4 rescue cell lines in LIF/serum. Relative expression values shown are the mean+/-SD of three biological replicates. (**B**) Immunostaining of additional knockout and Foxd4 rescue cell lines differentiated in N2B27+/-SB43. (**C**) Quantification of immunostaining of cells at day 5 of differentiation +/- 10 uM SB43 performed by nuclear segmentation and quantitative image analysis. Shown are the mean values for three independent biological replicates. A minimum of 8000 nuclei were scored per experiment. Data for all replicates are shown in Supplementary table 3. Scale bar: 50 μm

Table S1. CRISPR/Cas9 targeting guide RNA and ssODN template sequences.

Cell line generated	Component	Sequence
E2A-V5	Guide	AGCCGTCACAGCTTCTTCGT
	ssODN	GTCGGGGACCCACAGCTGGCCCTGTCAGCCGCCCA<br>CCCGGGCCTGGGTGAGGCCCACAACCCAGCCGGGC<br>ACCTGGGTAAGCCTATCCCTAACCCTCTCCTCGGTCT<br>CGATTCTACGTGAGCCGTCACAGCTTCTTCGTTGCAC<br>CAGCGACCACCATATCTCTGCCCGGGGTGCATCAGG<br>ACGGTTCTGGATGAG
E2A$^{-/-}$	Guide	TGCAAACTGGGTTCCCCCGA
	ssODN	CCTGACTTTTCTCTGTCCCCACAGATGTTCCCGCTAC<br>CAGTGGCCAATGGGAAGAGCCGGCCCGGCTCGCTC<br>GGGTGAGAATTCGGAACCCAGTTTGCAGGCTCAGGT<br>AGGACTGAGGGGCTCTTGGGGGGGTGTTCATGGGG<br>ACGGTGGCGGAGA
E2A$^{-/-}$HEB$^{-/-}$	Guide	CGGTTGAAATCGTCAGAATT
	ssODN	TATGTAAGTAATACATTTGAATTAATTATCCTGTTTTGT<br>TTTAAGGTATATGCACCATCCGCAAATTCTGACTAGG<br>AATTCCTTAAGTACCCATACGATGTTCCAGATTACGCT<br>GATTTCAACCGTGAATCTCCTAGTTACCCATCTCCCA<br>AGCCAGCAACCAGTATGTTCGCTAGCACTTTCTTTAT<br>GCAAGGTAAGC

Development • Supplementary information

Table S2. qRT-PCR primers and probes

Gene	Forward primer	Reverse primer	UPL probe
Cdh1 (E-cadherin)	ATCCTCGCCCTGCTGATT	ACCACCGTTCTCCTCCGTA	18
Cdh2 (N-cadherin)	GCCATCATCGCTATCCTTCT	CCGTTTCATCCATACCACAAA	18
Cripto (Tdgf1)	GTTTGAATTTGGACCCGTTG	GGAAGGCACAAACTGGAAAG	93
E2A	GTGGGCTCTGACAAGGAACT	ACAGGTAGCGGGAACATCAT	79
Eomes	ACCGGCACCAAACTGAGA	AAGCTCAAGAAAGGAAACATGC	9
Fgf5	AAAACCTGGTGCACCCTAGA	CATCACATTCCCGAATTAAGC	29
Foxd4	TGGAGATCAGACGGAAGAAGA	GATCGCTCCAGGCACTTATG	63
Id1	TCCTGCAGCATGTAATCGAC	GGTCCCGACTTCAGACTCC	78
Lefty2	GCCCTCATCGACTCTAGGC	AGCTGCTGCCAGAAGTTCAC	97

Development • Supplementary information

Nanog	CCTCCAGCAGATGCAAGAA	GCTTGCACTTCATCCTTTGG	25
Oct4	GTTGGAGAAGGTGGAACCAA	CTCCTTCTGCAGGGCTTTC	95
Pax3	AAAAGGCTAAACACAGCATCG	CAATATCGGAGCCTTCATCTG	110
Sox1	GTGACATCTGCCCCCATC	GAGGCCAGTCTGGTGTCAG	60
SDHA	CAGTTCCACCCCACAGGTA	TCTCCACGACACCCTTCTGT	71
T-brachyury	ACTGGTCTAGCCTCGGAGTG	TTGCTCACAGACCAGAGACTG	27

Development • Supplementary information

Table S3. Immunostaining quantification (associated with Figure 5)
(A) Cells differentiated in N2B27 **(B)** Cells differentiated in N2B27+SB43

(A)

-SB43	% Rep1	% Rep2	% Rep3	Mean %	Standard deviation
	**Parental**				
**Oct4-/Sox1+**	72.9	89.2	83.8	82.0	8.34
**Oct4+/Sox1-**	1.8	0.0	1.5	1.1	0.98
**Oct4+/Sox1+**	6.2	3.1	5.2	4.8	1.60
**Oct4-Sox1-**	19.1	7.7	9.5	12.1	6.13
	**E2A-/-**				
**Oct4-/Sox1+**	4.7	7.6	56.5	22.9	29.07
**Oct4+/Sox1-**	40.3	45.6	3.9	30.0	22.68
**Oct4+/Sox1+**	3.1	18.2	12.1	11.1	7.59
**Oct4-Sox1-**	51.9	28.6	27.5	36.0	13.76
	**E2A-/-HEB-/-**				
**Oct4-/Sox1+**	17.3	6.6	56.0	26.6	28.78
**Oct4+/Sox1-**	33.4	69.6	2.8	35.3	33.95
**Oct4+/Sox1+**	3.3	3.3	6.8	4.4	3.91
**Oct4-Sox1-**	46.1	20.5	34.4	33.7	14.03
	**E2A-/- +Foxd4 Cl.3**				
**Oct4-/Sox1+**	57.0	61.3	43.5	53.9	9.27
**Oct4+/Sox1-**	25.5	1.0	0.9	9.1	14.19
**Oct4+/Sox1+**	11.1	21.4	16.7	16.4	5.15
**Oct4-Sox1-**	6.4	16.3	38.9	20.5	16.64
	**E2A-/- +Foxd4 Cl.7**				
**Oct4-/Sox1+**	42.8	45.4	40.0	42.8	2.68
**Oct4+/Sox1-**	7.1	7.7	7.0	7.3	0.38
**Oct4+/Sox1+**	8.6	17.7	28.8	18.4	10.12
**Oct4-Sox1-**	41.5	29.1	24.1	31.5	8.96
	**E2A-/- +Foxd4 Cl.8**				
**Oct4-/Sox1+**	10.3	0.3	1.9	4.2	5.39
**Oct4+/Sox1-**	21.5	67.6	64.8	51.3	25.83
**Oct4+/Sox1+**	5.6	6.4	16.4	9.5	6.00
**Oct4-Sox1-**	62.5	25.8	16.9	35.1	24.19

Development • Supplementary information

(B)

+SB43	% Rep1	% Rep2	% Rep3	Mean %	Standard deviation
	Parental				
Oct4-/Sox1+	93.1	99.3	97.7	96.7	3.18
Oct4+/Sox1-	0.0	0.0	0.0	0.0	0.00
Oct4+/Sox1+	0.0	0.7	1.1	0.6	0.56
Oct4-Sox1-	6.9	0.0	1.2	2.7	3.66
	E2A-/-				
Oct4-/Sox1+	73.1	65.0	93.9	77.3	14.88
Oct4+/Sox1-	3.8	0.4	0.0	1.4	2.09
Oct4+/Sox1+	3.9	4.9	1.7	3.5	1.63
Oct4-Sox1-	19.2	29.7	4.4	17.8	12.70
	E2A-/-HEB-/-				
Oct4-/Sox1+	73.5	63.5	97.8	78.2	17.19
Oct4+/Sox1-	0.0	0.2	0.0	0.1	0.09
Oct4+/Sox1+	1.7	1.9	1.3	1.7	1.19
Oct4-Sox1-	24.7	34.4	0.9	20.0	16.92
	E2A-/- +Foxd4 Cl.3				
Oct4-/Sox1+	82.8	85.1	96.7	88.2	7.44
Oct4+/Sox1-	0.0	0.0	0.0	0.0	0.00
Oct4+/Sox1+	3.6	1.5	2.6	2.6	1.04
Oct4-Sox1-	13.5	13.4	0.6	9.2	7.40
	E2A-/- +Foxd4 Cl.7				
Oct4-/Sox1+	85.1	94.4	97.0	92.2	6.27
Oct4+/Sox1-	0.0	0.0	0.0	0.0	0.00
Oct4+/Sox1+	1.8	0.6	3.0	1.8	1.16
Oct4-Sox1-	13.1	5.0	0.0	6.0	6.60
	E2A-/- +Foxd4 Cl.8				
Oct4-/Sox1+	76.3	95.6	97.0	89.6	11.58
Oct4+/Sox1-	0.0	0.0	0.0	0.0	0.00
Oct4+/Sox1+	0.0	2.3	3.0	1.8	1.58
Oct4-Sox1-	23.7	2.1	0.0	8.6	13.14
