# Supplementary material for: The transcription factor E2A drives neural differentiation in pluripotent cells
Source: Development. 2020 Jun 22;147(12):dev184093. doi: 10.1242/dev.184093 (PMC7328008; doi:10.1242/dev.184093)
Supplement: Reviewer comments [file develop-147-184093_review_history.pdf]

# The transcription factor E2A drives neural differentiation in pluripotent cells

Chandrika Rao, Mattias Malaguti, John O. Mason and Sally Lowell
DOI: 10.1242/dev.184093

**Editor**: Francois Guillemot

**Review timeline**
Original submission: 21 August 2019
Editorial decision: 27 September 2019
First revision received: 28 April 2020
Accepted: 26 May 2020

---

**Original submission**

First decision letter

MS ID#: DEVELOP/2019/184093

MS TITLE: The transcription factor E2A drives neural differentiation in pluripotent cells

AUTHORS: Chandrika Rao, Mattias Malaguti, John Mason, and Sally Lowell

I have now received the reports of three referees on your manuscript and I have reached a decision. The reports are appended below and you can access them online. For this, please go to and click on the 'Manuscripts with Decisions' queue in the Author Area.

As you will see, all the referees express great interest in your work, but they also have significant criticisms and recommend a substantial revision of your manuscript before we can consider publication. If you are able to revise the manuscript along the lines suggested, which may involve further experiments, I will be happy to receive a revised version of the manuscript. The mutation of Cys residues in the E2A locus requested by referee 3 is an interesting experiment but it does not seem feasible during the revision period and is therefore not essential for the revision. Your revised paper will be re-reviewed by the original referees, and its acceptance will depend on your addressing satisfactorily all their major concerns. Please also note that Development will normally permit only one round of major revision.

Please attend to all of the reviewers' comments and ensure that you clearly highlight all changes made in the revised manuscript. Please avoid using 'Tracked changes' in Word files as these are lost in PDF conversion. I should be grateful if you would also provide a point-by-point response detailing how you have dealt with the points raised by the reviewers in the 'Response to Reviewers' box. If you do not agree with any of their criticisms or suggestions please explain clearly why this is so.

Reviewer 1

*Advance summary and potential significance to field*

This is a straightforward and logical study trying to fill in a gap in our knowledge of a regulatory step in neural specification; there has been prior identification of Id proteins as regulators of this process but why and how they work was largely obscure.Tthis paper shows that neural specification

© 2020. Published by The Company of Biologists under the terms of the Creative Commons Attribution License (https://creativecommons.org/licenses/by/4.0/). 

is regulated by E2A and this appears to be in the form of an E2A homodimer as opposed to its normal heterodimeric form. The knock-out data are pretty clear and show that E2A is required. The fact that E2A is acting as a homo-dimer largely rests on results generated from a forced homodimeric construct and mutants thereof, which is a slight concern; however, it is hard to see how the experiments can be done in another way short or knocking in hetero-dimer mutant forms of E2A into the endogenous locus, which would be beyond the norm. in the field and beyond the scope of this study.
This paper is self-contained, in that it sets out to ask a specific question and answers it. It opens up new areas, for instance why exactly the E2A knock-out blocks neural commitment but has a more complex effect on pluripotent genes and other lineages. However, in line with direction to Reviewers, one could view these sort of questions as the bases for follow-on studies rather than strictly necessary for publication of this work.

*Comments for the author*

In figures 1 and 2, E2A is measured by qPCR and there are immunostaining of cells but it would certainly be useful to have western blots of E2A to see what the level of protein is across the differentiation time-course. Does it mirror mRNA? Is the forced homodimer much more stable than the monomer and what about relative to the mutant homodimer? Can the authors rule out the fact that it is protein stability/accumulation rather than homodimerisation that leads to enhanced activity of the homodimer?
In Figure 3, I am confused about the Y axis. Relative expression? Relative to what as nothing is set at 1.
What do the numbers mean as they range from what seems like a tiny scale (0.00-0.02) with FoxD4 to up to 10 to the 15 with Fgf5. Should they just be relative to Serum/LIF? Are some of them detected att such a low level as to be barely detectable tho a fold-change may still be "significant". can the authors clarify?

A few things need to be better addressed in the discussion.
First, the authors are a rather narrow candidate view of how E2A might be working. As there is no ChIPseq data they should perhaps be clear that it may be having a multi-faceted effect and nodal and FoxD4 are just a couple of those possible facets. Also, is anything known about E boxes in the regulatory regions of Sox1 itself? Could E2A homodimers be acting directly on the Sox1 promoter? This should be discussed.
Finally, they should consider adding a note of caution; if the forces homodimer over-expression unregulates Id proteins but is not inhibited by it, then an endogenous negative feedback loop may not be active in their experimental scenario, which may be present normally. This should be discussed.

The study would be improved if they could show that neural differentiation induced by E2A hmodimers was resistant to Id protein over-expression or if Ids have an enhanced dose-dependent effect in E2A knock-out cells, although I acknowledge that this may also be considered beyond the scope of this study.

## Reviewer 2

*Advance summary and potential significance to field*

In this MS, Rao et al investigated a role for E2A in the earliest stages of mouse ESC neural differentiation. The group has previously identified the E2A gene product, E47 and E12, as the major binding partner of Id1, with Id1 a negative regulator of neural differentiation working downstream of BMP signalling. The authors hypothesized that the E2A homodimer is a key intrinsic regulator of neural fate commitment in mESCs.
The authors firstly examined E2A expression in undifferentiated ESCs and during neural differentiation by visualising V5 tag expression using an E2A-V5 knock-in mESC they generated. They then investigated the consequence of E2A gain- and loss-of-function in mESC differentiation. They show that enforced expression of E2A homodimer promote neural fate as evidenced by Sox1 induction. In contrast E2A homozygous knockout cells exhibit defect in neural induction. They also performed bulk RNAseq between the parental and E2A overexpressing cells. By analysing the

© 2020. Published by The Company of Biologists under the terms of the Creative Commons Attribution License (https://creativecommons.org/licenses/by/4.0/).

RNAseq data the authors identified Nodal signalling and Foxd4 as candidate early response signalling/genes of E2A and carried out rescue experiment using small molecule inhibition of Nodal signalling and transgene expression of Foxd4 in E2A mutant cells, alone and in combination.
The MS presents a substantial body of original work and is clearly written with convincing data. I have no major concerns but two minor issues.
1, the authors identify neural fate almost exclusively based on Sox1 expression, the conclusions would be stronger if they provide more evidence on neural identity at later points (eg. neuronal marker expression).
2, for readers interested in the human system it would be helpful to see discussions whether their findings might be generalized to the human cells.

*Comments for the author*

see above

Reviewer 3

*Advance summary and potential significance to field*

This paper by Rao et al addresses important questions regarding the early transcriptional mechanisms that drive acquisition of neural fate from pluripotent stem cells which is currently only fragmentarily understood. The author propose that the bHLH transcription factor E2A plays an important role in this process. They show that expression of a forced E2A homodimer promotes neural fate in mouse embryonic stem cells and that knockout of E2A together with another E-protein HEB impairs neural fate which can be rescued by forced expression of downstream targets (as identified by RN-seq) Foxd4 and repression of Nodal signaling. This data are interesting, but as detailed below the expression of forced E2A dimers should be complemented by an additional experiment which would strengthen the view that E2A homodimers play an essential role.

*Comments for the author*

The key experiment I would suggest is to replace the wildtype E2A locus with one in which the amino acid required for disulfide bridges is mutated. This would go beyond the regular ko and reveal whether it is indeed the homodimers which account for the early acquisition of neural fate as read out by Sox1. This would raise in my view the relevance of the study. In its current version, the main argument of the paper for supporting the role of E2A homodimers is based on an artificial situation where E2A proteins are chained.
In this context, the authors should provide new evidence or discuss in more detail for the mentioned fact that heterodimerisation with other bHLH factors does not depend on the cystein residues.
Also, in the mutant experiments, cystein residues in both chained E2A proteins are mutated. I wonder whether logically one mutant AA should not be enough to render the stabilisation impossible. (As an aside, are the chained E2A proteins still capable of interacting with additional bHLH proteins?)

Minor points:
1. Ref Di-Gregorio seems missing in the REF list; see also Lin and Tatar, in prep.
2. Figure 1: The V5 staining is not very impressive. Higher magnification images should be shown. Also, V5 IR should be backed by western blot. The notion of a dynamic expression of E2A is not really supported by the IF data.
3. Also here, higher magnification images would help to illustrate Sox1. By the way, E cadherin RNA goes down in D. Later the manuscript states that E-cadherin does not seem to be downstream of E2A expression. This refers to protein. But as such the statement seems to be too strong.
3. What is the regional identity of the neural cells derived from ES cells? Hind brain? (Pax3). This might be important as to whether Foxd4 is region specific or not. In other words, is the role of E2A proteins proposed here limited to specific regional identities within the CNS?
4. For non-E2A-aficionados it would be useful to explain how E2A relate to E12 and E47 and what the chained E2A proteins represent with regard to these two transcripts.
5. Page 5: Tuj1 is a neuronal marker but not of mature neurons

© 2020. Published by The Company of Biologists under the terms of the Creative Commons Attribution License (https://creativecommons.org/licenses/by/4.0/). 

6. SB431542 is not specific to Nodal pathway. Could this be back up with a more specific manipulation?

---

**First revision**

Author response to reviewers' comments

**Response to reviewers**
DEVELOP/2019/184093
The transcription factor E2A drives neural differentiation in pluripotent cells
Chandrika Rao, Mattias Malaguti, John Mason, and Sally Lowell

Reviewer 1 Advance Summary and Potential Significance to Field:

This is a straightforward and logical study trying to fill in a gap in our knowledge of a regulatory step in neural specification; there has been prior identification of Id proteins as regulators of this process but why and how they work was largely obscure. This paper shows that neural specification is regulated by E2A and this appears to be in the form of an E2A homodimer as opposed to its normal heterodimeric form. The knock-out data are pretty clear and show that E2A is required. The fact that E2A is acting as a homo- dimer largely rests on results generated from a forced homodimeric construct and mutants thereof, which is a slight concern; however, it is hard to see how the experiments can be done in another way short or knocking in hetero-dimer mutant forms of E2A into the endogenous locus, which would be beyond the norm in the field and beyond the scope of this study.
This paper is self-contained, in that it sets out to ask a specific question and answers it. It opens up new areas, for instance why exactly the E2A knock-out blocks neural commitment but has a more complex effect on pluripotent genes and other lineages. However, in line with direction to Reviewers, one could view these sort of questions as the bases for follow-on studies rather than strictly necessary for publication of this work.

Reviewer 1 Comments for the Author:
In figures 1 and 2, E2A is measured by qPCR and there are immunostaining of cells but it would certainly be useful to have western blots of E2A to see what the level of protein is across the differentiation time- course. Does it mirror mRNA? Is the forced homodimer much more stable than the monomer and what about relative to the mutant homodimer? Can the authors rule out the fact that it is protein stability/accumulation rather than homodimerisation that leads to enhanced activity of the homodimer?

> In our revised manuscript we include new western blot data in Fig 1C showing that E2A protein expression does indeed mirror the mRNA expression show in our qPCR data. E2A mRNA and protein are both expressed stably during the neural differentiation timecourse with a slight increase over time. We have discussed this within the text (lines 90-93).
>
> We also include new western blot data in Supp Fig 3C showing that expression of the forced homodimer is considerably lower than expression of the monomer. This rules out the possibility that it is protein stability/accumulation rather than homodimerisation that leads to enhanced activity of the homodimer. We have discussed this within the text (lines 132-136).
>
> Our new data in Supp Fig 3C also shows that of the two clones of mutant homodimer cells are expressed at slightly lower levels than the wild type homodimer. We have discussed this within the text (lines 136-137).

In Figure 3, I am confused about the Y axis. Relative expression? Relative to what as nothing is

© 2020. Published by The Company of Biologists under the terms of the Creative Commons Attribution License (https://creativecommons.org/licenses/by/4.0/). 

set at 1. What do the numbers mean as they range from what seems like a tiny scale (0.00-0.02) with FoxD4 to up to 10 to the 15 with Fgf5. Should they just be relative to Serum/LIF? Are some of them detected at such a low level as to be barely detectable though a fold-change may still be "significant". Can the authors clarify?

We apologise for not making this clear in our original manuscript. Expression values shown are relative to the mean expression values of the housekeeping gene Sdha. This was previously mentioned only within the methods section and we agree it would be better to make this clear on the figure. We have now re-labelled the Y-axis of these graphs to state "Relative to Sdha"

A few things need to be better addressed in the discussion.
First, the authors are a rather narrow candidate view of how E2A might be working. As there is no ChIPseq data they should perhaps be clear that it may be having a multi-faceted effect and nodal and FoxD4 are just a couple of those possible facets. Also, is anything known about E boxes in the regulatory regions of Sox1 itself? Could E2A homodimers be acting directly on the Sox1 promoter? This should be discussed.
Finally, they should consider adding a note of caution; if the forces homodimer over-expression upregulates Id proteins but is not inhibited by it, then an endogenous negative feedback loop may not be active in their experimental scenario, which may be present normally. This should be discussed.

We agree that E2A is likely to have a multi-faceted effect. Studies in Xenopus (Wills & Baker 2015) indicate that E2A regulates a relatively large network of TFs. We have expanded our discussion on this point to state "It is likely that E2A has multiple downstream effectors in mammalian cells, as it does in Xenopus (Wills & Baker 2015) but our rescue experiments suggest that inhibition of nodal and activation of Foxd4 are key effectors that explain the ability of E2A to drive neural differentiation" (lines 337-340).

We do not know whether E2A homodimers directly bind to the regulatory elements of Sox1: the presence of E-boxes is not a reliable indicator of E2A binding and Chip-seq experiments would be required.

We have added the following sentence to the discussion to make it clear that forced homodimerisation of E2A may disrupt an Id-mediated negative feedback loop: "We note that our forced homodimerisation of E2A is likely to disrupt this negative feedback loop, and that this effect may help amplify the pro-neural activity of E2A homodimers" (lines 298-299). However we note that this effect alone is unlikely to be central to explaining the pro-neural effects of E2A because the E2A null phenotype can be rescued by combined inhibition of nodal and activation of Foxd3, suggesting that these are key downstream mediators of E2A's proneural activity (although of course this does not exclude other contributory mechanisms).

The study would be improved if they could show that neural differentiation induced by E2A homodimers was resistant to Id protein over-expression or if Ids have an enhanced dose-dependent effect in E2A knock-out cells, although I acknowledge that this may also be considered beyond the scope of this study.

These would be interesting experiments. We do think they are beyond the scope of the current study but we have added a new section to the discussion to discuss how our model could be further tested by asking whether E2A homodimers render neural differentiation resistant to the inhibitory effects of Id proteins (lines 290-292).

Reviewer 2 Advance Summary and Potential Significance to Field:
In this MS, Rao et al investigated a role for E2A in the earliest stages of mouse ESC neural differentiation. The group has previously identified the E2A gene product, E47 and E12, as the major binding partner of Id1, with Id1 a negative regulator of neural differentiation working downstream of BMP signalling. The authors hypothesized that the E2A homodimer is a key

© 2020. Published by The Company of Biologists under the terms of the Creative Commons Attribution License (https://creativecommons.org/licenses/by/4.0/). 

intrinsic regulator of neural fate commitment in mESCs.
The authors firstly examined E2A expression in undifferentiated ESCs and during neural differentiation by visualising V5 tag expression using an E2A-V5 knock-in mESC they generated. They then investigated the consequence of E2A gain- and loss-of-function in mESC differentiation. They show that enforced expression of E2A homodimer promote neural fate as evidenced by Sox1 induction. In contrast, E2A homozygous knockout cells exhibit defect in neural induction. They also performed bulk RNAseq between the parental and E2A overexpressing cells. By analysing the RNAseq data the authors identified Nodal signalling and Foxd4 as candidate early response signalling/genes of E2A and carried out rescue experiment using small molecule inhibition of Nodal signalling and transgene expression of Foxd4 in E2A mutant cells, alone and in combination.

Reviewer 2 Comments for the Author:
The MS presents a substantial body of original work and is clearly written with convincing data. I have no major concerns but two minor issues.

1, the authors identify neural fate almost exclusively based on Sox1 expression, the conclusions would be stronger if they provide more evidence on neural identity at later points (e.g. neuronal marker expression).

> We include data in Fig 3B and 3D showing that E47 is required for upregulation of early neural markers Pax3, FoxD4 and early neuronal marker TUJ1 and that is required for efficient repression of non-neural markers T-Brachyury & Eomes. We also show in Fig 4 that E47 homodimers upregulate neural markers Neurog3, NeuroD4, FoxD4 and Id2. Our GO analysis also supports the conclusion that E47 homodimers specifically upregulate genes related to neural development and not other lineages. We therefore feel confident that E47 homodimers activate key aspects of the early neural differentiation programme even under non-permissive differentiation conditions. However we should emphasise that we do not claim that E2A homodimers can dominantly generate fully functional neurons, and indeed we think it likely that other factors would be required in order to fully progress through later stages of neuronal differentiation in the presence of anti-neural signals. This is discussed in lines 205-208.

2, for readers interested in the human system it would be helpful to see discussions whether their findings might be generalized to the human cells.

> Thank you for this suggestion. We have added the following statement to the discussion (lines 334-337) "Given that inhibition of Nodal also promotes neural differentiation of human pluripotent cells (Chambers et al 2009), and that E2A regulates lefty in human cells (Yoon et al 2011) it seems likely that E2A may also drive neural differentiation of human pluripotent cells, although this remains to be tested."

Reviewer 3 Advance Summary and Potential Significance to Field:
This paper by Rao et al addresses important questions regarding the early transcriptional mechanisms that drive acquisition of neural fate from pluripotent stem cells which is currently only fragmentarily understood. The authors propose that the bHLH transcription factor E2A plays an important role in this process. They show that expression of a forced E2A homodimer promotes neural fate in mouse embryonic stem cells and that knockout of E2A together with another E-protein HEB impairs neural fate which can be rescued by forced expression of downstream targets (as identified by RN-seq) Foxd4 and repression of Nodal signalling. This data are interesting, but as detailed below the expression of forced E2A dimers should be complemented by an additional experiment, which would strengthen the view that E2A homodimers play an essential role.

Reviewer 3 Comments for the Author:
The key experiment I would suggest is to replace the wildtype E2A locus with one in which the amino acid required for disulfide bridges is mutated. This would go beyond the regular ko and reveal whether it is indeed the homodimers which account for the early acquisition of neural fate as read out by Sox1. This would raise in my view the relevance of the study. In its current

© 2020. Published by The Company of Biologists under the terms of the Creative Commons Attribution License (https://creativecommons.org/licenses/by/4.0/).

version, the main argument of the paper for supporting the role of E2A homodimers is based on an artificial situation where E2A proteins are chained.
In this context, the authors should provide new evidence or discuss in more detail for the mentioned fact that heterodimerisation with other bHLH factors does not depend on the cysteine residues.
Also, in the mutant experiments, cysteine residues in both chained E2A proteins are mutated. I wonder whether logically one mutant AA should not be enough to render the stabilisation impossible. (As an aside, are the chained E2A proteins still capable of interacting with additional bHLH proteins?)

We agree that these would be interesting experiments, but we suggest that they are beyond the scope of the current study. Our revised discussion now discusses the fact that C57A mutation of endogenous E2A will be an important for testing whether homodimerisation of endogenous E2A is required for efficient neural differentiation (line 290-293).
Tethered E2A proteins with a C57A mutation are still able to heterodimerise with for example MyoD but are unable to homodimerise (Benezra 1994): this is discussed in our revised manuscript (line 127-128).

Minor points:
1. Ref Di-Gregorio seems missing in the REF list; see also Lin and Tatar, in prep.

We have added the missing reference to Di-Gregorio et al. Lin, Tatar et al is now deposited on BioRxiv and this preprint has been added to the reference list.

2. Figure 1: The V5 staining is not very impressive. Higher magnification images should be shown. Also, V5 IR should be backed by western blot. The notion of a dynamic expression of E2A is not really supported by the IF data.

In our revised manuscript we include new western blot data in Fig 1C showing that E2A protein expression does indeed mirror the mRNA expression show in our qPCR data. E2A mRNA and protein are both expressed stably during the neural differentiation timecourse with a slight increase over time. We have discussed this within the text ((lines 90-93).

We also provide higher magnification images in the revised version of Fig 1.

3. Also here, higher magnification images would help to illustrate Sox1. By the way, E cadherin RNA goes down in D. Later the manuscript states that E-cadherin does not seem to be downstream of E2A expression. This refers to protein. But as such the statement seems to be too strong.

We provide higher magnification images in Fig2B.

We have modified our conclusions about E-Cadherin, which now state: "Our findings do not exclude the possibility that E-Cadherin is a transcriptional target of E2A in pluripotent cells, but they do suggest that E-Cadherin is unlikely to mediate effects of E2A on neural differentiation." (lines 180-182).

3. What is the regional identity of the neural cells derived from ES cells? Hind brain? (Pax3). This might be important as to whether Foxd4 is region specific or not. In other words, is the role of E2A proteins proposed here limited to specific regional identities within the CNS?

This remains an open question. Our RNAseq data on E2A-responsive genes identifies genes characteristic of early neural identity but does not clearly identify any particular regional identity. We are reluctant to draw any conclusions from this given that our analysis was carried out only at relatively early time points.

© 2020. Published by The Company of Biologists under the terms of the Creative Commons Attribution License (https://creativecommons.org/licenses/by/4.0/).

4. For non-E2A-aficionados it would be useful to explain how E2A relate to E12 and E47 and what the chained E2A proteins represent with regard to these two transcripts.

In our revised manuscript we state that E12/E47 are alternatively spliced gene products of E2A (line 37). The main text (line 107) and methods (lines 391-397) state that the monomer and forced homodimers we use in this study are comprised of E47 sequences.

5. Page 5: Tuj1 is a neuronal marker but not of mature neurons

We have clarified this point in the text (line 162).

6. SB431542 is not specific to Nodal pathway. Could this be back up with a more specific manipulation?

SB43 is a relatively specific inhibitor of Tgfb signalling insofar as it not affect BMP/ERK/JNK/MAPK signalling (Inman et al 2002) but we agree with the reviewer that we cannot exclude effects through other members of the Tgfb superfamily that operate through ALK4,5 or 7. We have modified the text throughout the paragraph describing these experiments (lines 238-246) to replace "Nodal" with " TGF-β" and have added reference to Inman et al (line 239)

**References**

**Chambers, S.M., Fasano, C.A., Papapetrou, E.P., Tomishima, M., Sadelain, M., and Studer, L.** (2009). Highly efficient neural conversion of human ES and iPS cells by dual inhibition of SMAD signaling. *Nat. Biotechnol.* **27**, 275–280.

Inman, G.J., Nicolas, F.J., Callahan, J.F., et al. (2002). SB-431542 is a potent and specific inhibitor of transforming growth factor-b superfamily type I activin receptor-like kinase (ALK) receptors ALK4, ALK5, and ALK7. Mol. Pharmacol. 62, 65–74.

Wills, A.E., and Baker, J.C. (2015). E2a Is Necessary for Smad2/3-Dependent Transcription and the Direct Repression of lefty during Gastrulation. *Developmental Cell* **32**, 345–357.

Yoon, S.-J., Wills, A.E., Chuong, E., Gupta, R., and Baker, J.C. (2011). HEB and E2A function as SMAD/FOXH1 cofactors. *Genes Dev.* **25**, 1654–1661.

---

Second decision letter

MS ID#: DEVELOP/2019/184093

MS TITLE: The transcription factor E2A drives neural differentiation in pluripotent cells

AUTHORS: Chandrika Rao, Mattias Malaguti, John Mason, and Sally Lowell
ARTICLE TYPE: Research Article

I am delighted to tell you that your manuscript has been accepted for publication in Development, pending our standard ethics checks.

Reviewer 3

© 2020. Published by The Company of Biologists under the terms of the Creative Commons Attribution License (https://creativecommons.org/licenses/by/4.0/). 

*Advance summary and potential significance to field*

The authors provide compelling evidence that E2A plays an important role in inducing neural identity. As the paper does not make the claim that this function is mediated by E2A homodimers, I agree that the suggested experiment, in which E2A WT is replaced by a version that does not encode the critical cystein, may go beyond the scope of the study. The remainder issues were adequately addressed.

*Comments for the author*

N/A

© 2020. Published by The Company of Biologists under the terms of the Creative Commons Attribution License (https://creativecommons.org/licenses/by/4.0/).
